# Nanogel-mediated delivery of oncomodulin secreted from regeneration-associated macrophages promotes sensory axon regeneration in the spinal cord

**DOI:** 10.7150/thno.73386

**Published:** 2022-08-01

**Authors:** Min Jung Kwon, Yeojin Seo, Hana Cho, Hyung Soon Kim, Young Joo Oh, Simay Genişcan, Minjae Kim, Hee Hwan Park, Eun-Hye Joe, Myung-Hee Kwon, Han Chang Kang, Byung Gon Kim

**Affiliations:** 1 Department of Brain Science, Ajou University Graduate School of Medicine, Suwon, 16499, Republic of Korea.; 2 Neuroscience Graduate Program, Department of Biomedical Sciences, Ajou University Graduate School of Medicine, Suwon, 16499, Republic of Korea.; 3 Department of Pharmacy, College of Pharmacy, The Catholic University of Korea, Bucheon, 14662, Republic of Korea.; 4 Department of Microbiology, Ajou University School of Medicine, Suwon 16499, Republic of Korea.; 5 Department of Pharmacology, Ajou University School of Medicine, Suwon, 16499, Republic of Korea.; 6 Center for Convergence Research of Neurological Disorders, Ajou University School of Medicine, Suwon, 16499, Republic of Korea.; 7 Department of Neurology, Ajou University School of Medicine, Suwon, 16499, Republic of Korea.; 8 AI-Superconvergence KIURI Translational Research Center, Suwon, 16499, Republic of Korea.

**Keywords:** Oncomodulin, Axon regeneration, Nanogel, Spinal cord injury, Regeneration-associated macrophages

## Abstract

Preconditioning nerve injury enhances axonal regeneration of dorsal root ganglia (DRG) neurons in part by driving pro-regenerative perineuronal macrophage activation. How these macrophages influence the neuronal capacity of axon regeneration remains elusive. We report that oncomodulin (ONCM) is produced from the regeneration-associated macrophages and strongly influences regeneration of DRG sensory axons. We also attempted to promote sensory axon regeneration by nanogel-mediated delivery of ONCM to DRGs.

**Methods:** *In vitro* neuron-macrophage interaction model and preconditioning sciatic nerve injury were used to verify the necessity of ONCM in preconditioning injury-induced neurite outgrowth. We developed a nanogel-mediated delivery system in which electrostatic encapsulation of ONCM by a reducible epsilon-poly(_L_-lysine)-nanogel (REPL-NG) enabled a controlled release of ONCM.

**Results:** Sciatic nerve injury upregulated ONCM in DRG macrophages. ONCM in macrophages was necessary to produce pro-regenerative macrophages in the *in vitro* model of neuron-macrophage interaction and played an essential role in preconditioning-induced neurite outgrowth. ONCM increased neurite outgrowth in cultured DRG neurons by activating a distinct gene set, particularly neuropeptide-related genes. Increasing extracellularly secreted ONCM in DRGs sufficiently enhanced the capacity of neurite outgrowth. Intraganglionic injection of REPL-NG/ONCM complex allowed sustained ONCM activity in DRG tissue and achieved a remarkable long-range regeneration of dorsal column sensory axons beyond spinal cord lesion.

**Conclusion:** NG-mediated ONCM delivery could be exploited as a therapeutic strategy for promoting sensory axon regeneration following spinal cord injury.

## Introduction

Axon regeneration failure following CNS injury is in part due to poor intrinsic capacity of axonal growth in mature CNS neurons 1. In contrast, PNS neurons possess the ability to activate axon growth programs upon injury. In a conditioning injury model, preceding injury to the sciatic nerve dramatically enhances the capacity of sensory neurons in the dorsal root ganglia (DRGs) and enables the DRG sensory axons to achieve some degree of regeneration following a subsequent injury to the spinal cord dorsal column 2, 3. Many studies attempted to explain how the preconditioning injury can boost the intrinsic regeneration potential of DRG neurons, but the precise cellular and molecular mechanisms mediating the conditioning effects remain elusive 4. Most of the previous studies have focused on DRG neuronal cells to identify changes in intracellular signaling, transcription factor activation, epigenetic landscape, and metabolic profiles in DRG neurons 5-11. In contrast, relatively little attention has been paid to non-neuronal cells surrounding DRG neurons. Recent studies suggest an intriguing possibility that these non-neuronal cells may also contribute to the enhanced regenerative capacity of DRG sensory neurons induced by the preconditioning injury 4, 12-15.

Several studies reported that peripheral nerve injury leads to activation of macrophages in DRGs and that the perineuronal macrophages in turn can contribute to the increase in the capacity of axonal growth in DRG sensory neurons 16-18. Axotomized DRG neurons and surrounding macrophages are highly likely to interact with each other to transmit injury signals from neurons to macrophages and to provide factors to stimulate axon regeneration from the regeneration-associated macrophages (RAMs) to neurons 19, 20. The neuron-macrophage interaction was recapitulated in an *in vitro* system in which a conditioned medium obtained from neuron-macrophage co-cultures treated with cAMP provides potent neurite outgrowth activities 16. Employing this neuron-macrophage interaction model, we previously demonstrated that preconditioned DRG neurons generate CCL2/CCR2 chemokine signaling to recruit and activate perineuronal macrophages 19, 21. However, it remains to be studied how these RAMs exert their effects on neurons in return to activate axon growth programs.

Oncomodulin (ONCM) was identified as a myeloid cell-derived molecule that supports the regeneration of retinal ganglion neurons following optic nerve injury 22, 23. However, the ability of ONCM to promote axonal regeneration outside of the retina has not been reported. Here, we identify ONCM as an effector molecule derived from RAMs that are activated by the neuron-macrophage interaction. ONCM expression is increased in DRG macrophages following preconditioning injury in a CCR2-dependent manner. The ONCM upregulation is required for the *in vitro* neuron-macrophage interaction and preconditioning-induced enhancement of axon growth capacity. Furthermore, we demonstrate that localized delivery of ONCM using a nano-scale hydrogel system, which was recently invented for efficient intracellular delivery of various molecules, peptide drugs in particular 24-26, into DRGs supports a highly robust extent of sensory axon regeneration well beyond the spinal injury site, implicating ONCM-encapsulated nanogel as a potential therapeutic strategy to promote axonal regeneration following CNS injury.

## Results

### Expression of ONCM in DRGs is required for preconditioning effects

**Figure 1:**
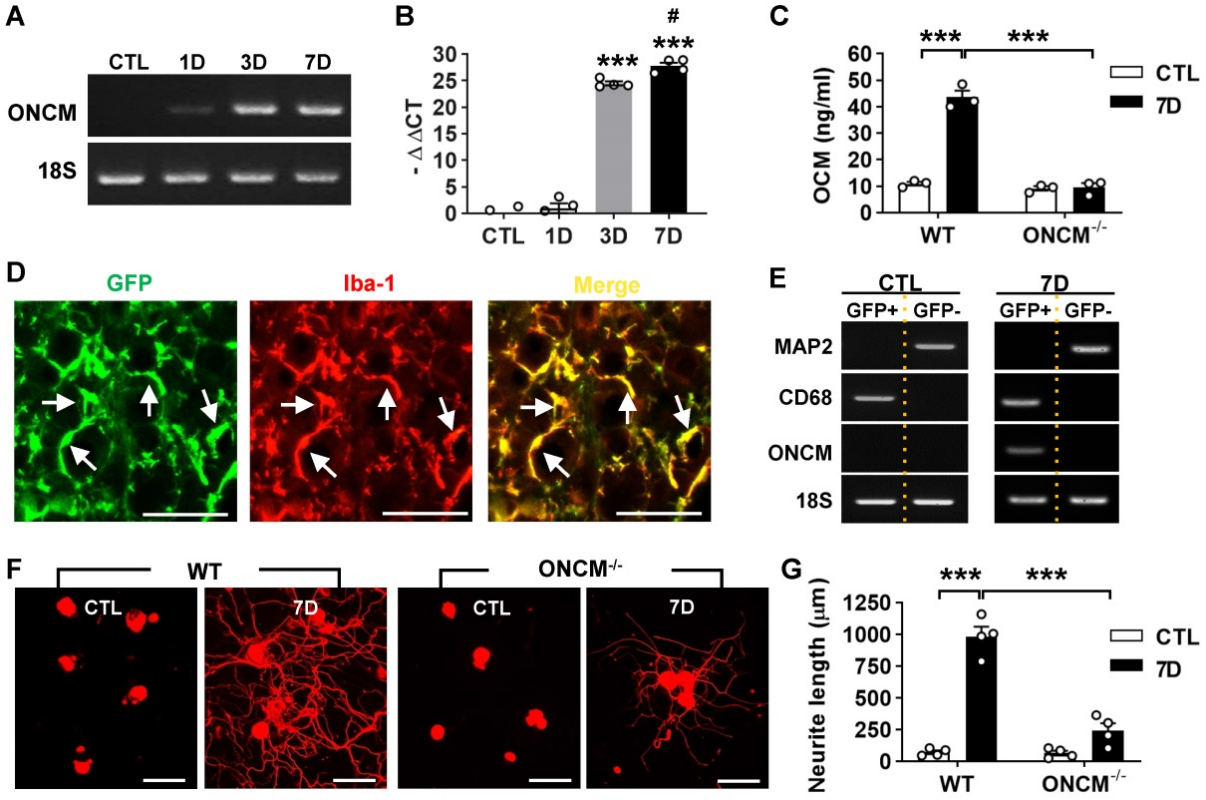
** Expression of oncomodulin in DRG macrophages is required for preconditioning effects. A,** Representative images of electrophoresed RT-PCR products for oncomodulin (ONCM). 18S rRNA was used as an internal reference. L4 and L5 DRGs were obtained 1, 3, and 7 d after ipsilateral sciatic nerve injury (SNI). **B,** Comparison of ONCM mRNA levels measured by quantitative RT-PCR. N = 4 animals for each time point. ***p < 0.001 compared with CTL and 1D time points and #*p* <0.05 compared with 3D time point by one-way ANOVA, followed by Tukey's *post hoc* analysis. **C,** ELISA measurement of ONCM levels in DRGs from WT and ONCM^-/-^ mice at 0 (CTL) and 7 d after SNI. N = 3 animals for each group. ***p < 0.001 compared with CTL values by unpaired *t* test. **D,** Representative immunofluorescence images of L5 DRG tissue sections obtained from Cx3cr1-GFP mice at 7 d after SNI. Tissue sections were stained with anti-GFP (green) and anti Iba-1 (red) antibodies. Arrows indicate cells positive for both markers. Scale bars represent 100 µm. **E,** Representative RT-PCR analysis of MAP2, CD68, and ONCM gene expressions from DRG cell fractions separated by FACS. L3, L4, and L5 DRGs from Cx3cr1-GFP mice at 0 (CTL) and 7 d after SNI. MAP2-positive neurons were isolated in the GFP negative fractions. CD68 and ONCM-positive macrophages were collected in the GFP positive fractions. **F,** Representative images of neurite outgrowth of DRG neurons taken from WT and ONCM^-/-^ mice 0 (CTL) and 7 d after SNI. DRG neurons from the L3, L4, and L5 DRGs were dissociated and cultured for 15 h before being fixed for the immunofluorescent visualization of neurites with anti-beta3 tubulin. Scale bars represent 100 µm. **G,** Comparison of the mean neurite length between cultures obtained from WT and ONCM^-/-^ mice at 0 (CTL) and 7 d after SNI. N = 4 animals for each group. **p < 0.01 by unpaired *t* test.

ONCM gene expression was examined in the lumbar DRGs following preconditioning sciatic nerve injury (SNI). ONCM mRNA level increased at 3- and 7-days post-injury (dpi) by more than 20 folds (Figure 1A-B). ELISA measurement of DRG lysates also revealed a marked increase in ONCM protein level at 7 dpi (Figure 1C). There was no detectable increase in ONCM protein at 7 dpi in ONCM-deficient mice. To ascertain that ONCM is expressed exclusively in macrophages, DRG macrophages were FACS-isolated using Cx3cr1-GFP transgenic mice. The number of perineuronal GFP positive cells sharply increased following SNI (Figure S1A), and all the GFP positive cells were colocalized with Iba-1 immunoreactivity (Figure 1D). GFP positive cells were readily separated from GFP negative fractions using flow cytometry (Figure S1B). MAP2, a neuron-specific gene, was expressed only in GFP negative fraction, and macrophage specific CD68 was expressed exclusively in GFP positive fraction, indicating a successful separation of the macrophage fraction. ONCM mRNA expression was detected only in GFP positive fraction from 7 dpi animals (Figure 1E), indicating that SNI-induced ONCM upregulation in DRGs occurs exclusively in perineuronal macrophages surrounding DRG neurons.

Next, we asked whether ONCM expression is necessary for the conditioning effects where preconditioning nerve injury enhances the capacity of axon growth in DRG sensory neurons 2, 3. Preconditioning SNI was conducted and the lumbar DRGs were dissected out at 7 dpi for neurite outgrowth assay. The SNI resulted in an increase in neurite outgrowth in wild type (WT) animals (Figure 1F-G). However, the preconditioning effects on neurite outgrowth were markedly attenuated in ONCM-deficient mice (Figure 1F-G). Our previous study showed that macrophage activation is essential for maintaining enhanced regenerative capacity, but dispensable for early induction of the preconditioning effects 16. When lumbar DRG neurons were acutely cultured only at 2 dpi, neurite outgrowth was still increased to an appreciable extent (Figure S2A). The enhanced neurite outgrowth activity was not abrogated in ONCM-deficient mice as opposed to a marked reduction of neurite length in ONCM-deficient neurons at 7 dpi (Figure S2A-B), indicating that macrophage-derived ONCM is not involved in inducing preconditioning effects. The intensity of Iba-1 immunoreactivity at the injury site in the sciatic nerve was comparable between WT and ONCM-deficient mice (Figure S3A). Furthermore, the number of Iba-1 positive perineuronal macrophages and the intensity of Iba-1 immunoreactivity in DGRs were not appreciably different between WT and ONCM-deficient mice (Figure S3B), indicating that activation of perineuronal macrophages per se was not affected by ONCM deficiency.

### Macrophage-derived ONCM is necessary for *in vitro* neuron-macrophage interaction

**Figure 2:**
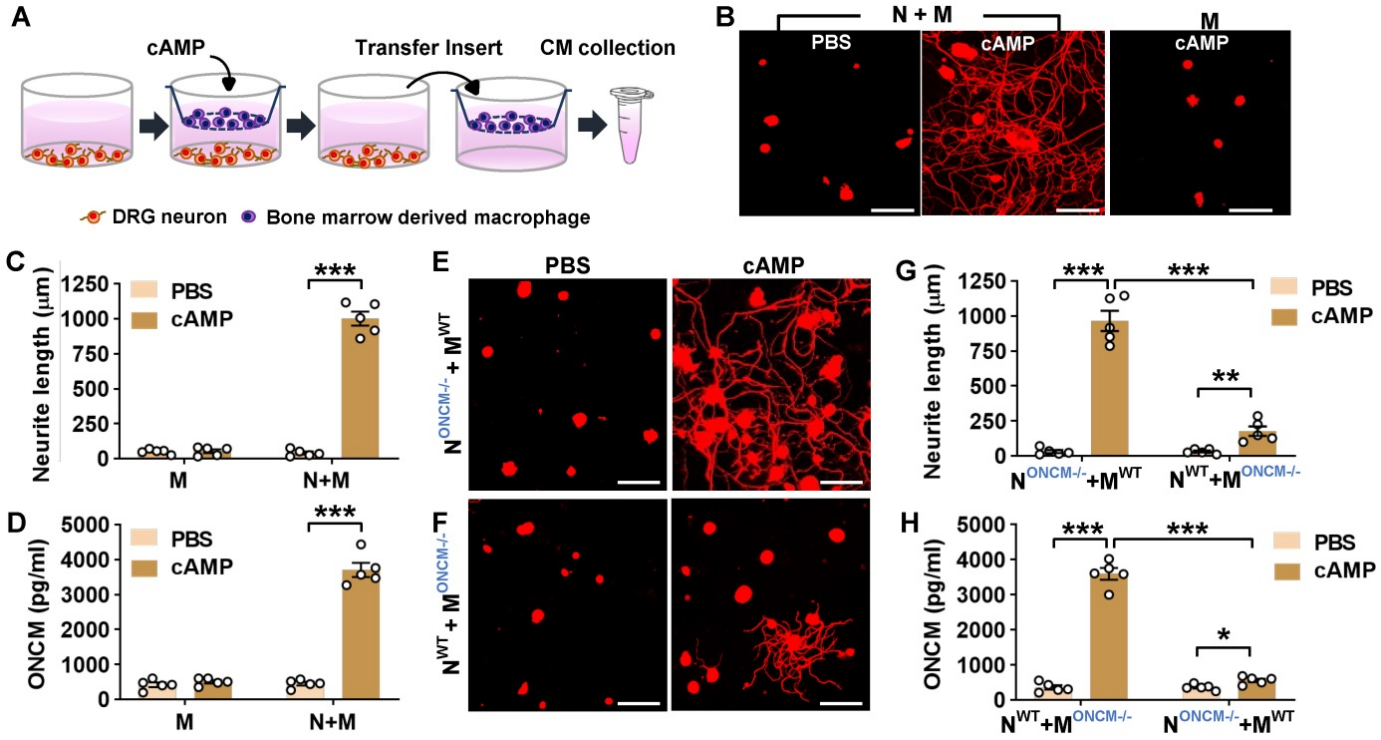
** Macrophage-derived ONCM is necessary for *in vitro* neuron-macrophage interaction. A,** Schematic diagram illustrating procedures to obtain macrophage conditioned medium (MCM) containing neurite outgrowth activity. Adult DRG neurons (1 x 10^6^ cells per well) are cultured for 4 h, and bone-marrow derived macrophages (5 x 10^6^ cells per well) are cultured on a cell culture insert. At 4 h after plating, either PBS or dibutyryl cAMP (db-cAMP) is treated. After 24 h incubation, the insert is transferred to another well containing about 1 ml fresh culture medium to collect MCM for 72 h. **B,** Representative results of neurite outgrowth assay using MCM obtained from various conditions. Cultured DRG neurons and their neurites were visualized by immunofluorescence staining for beta3 tubulin. Scale bars present 100 µm. **C,** Quantification graph comparing the mean neurite length. N = 4 independent cultures using independent MCMs for each condition. ***p < 0.001 by unpaired *t* test. **D,** ELISA measurement of the ONCM concentration in the MCM obtained from different culture conditions. N+M, Neuron-macrophage co-cultures; M, macrophage-only cultures. N = 4 independent cultures using independent MCMs for each condition. ***p < 0.001 c by unpaired *t* test. **E, F,** Representative images of neurite outgrowth in DRG neuron cultures treated with MCM obtained from neuron-macrophage co-cultures using WT or ONCM-deficient (ONCM^-/-^) neurons (N) or macrophages (M). All co-cultures were treated with either PBS or db-cAMP (cAMP), and the CMs were collected for 72 h. DRG neurons and their neurites were visualized by immunofluorescence staining for beta3 tubulin. Scale bars represent 100 µm. **G,** Quantification graph of neurite outgrowth in the presence of MCM from cultures of the different neuron-macrophage genotype combinations treated with either PBS or cAMP. N = 4 independent cultures using independent MCMs for each condition. ***p < 0.001 and **p < 0.01 by unpaired *t* test. **H,** ELISA measurement of the ONCM concentration in the cell culture media obtained from different neuron-macrophage genotype combinations treated with PBS or cAMP. N = 4 independent cultures using independent MCMs for each condition. ***p < 0.001 and *p < 0.05 by unpaired *t* test.

In the *in vitro* neuron-macrophage interaction model, peritoneal macrophages were co-cultured with DRG sensory neurons, and macrophage conditioned medium (MCM) collected from the co-cultures with cAMP treatment exhibited robust neurite outgrowth activity 27. In the current study, bone marrow-derived macrophages (BMDMs) were used because BMDMs tend to produce a higher number of cells and maintain a more stable morphology than peritoneal macrophages. BMDMs were co-cultured with DRG neurons for 24 h being treated with or without cAMP and then separated from the co-cultured neurons to collect MCM for 72 h thereafter (Figure 2A). MCM collected from the co-cultured BMDMs with cAMP showed robust neurite outgrowth activity (Figure 2B-C). However, MCM collected from BMDM only culture treated with cAMP did not show significant neurite outgrowth activity (Figure 2B-C), confirming that neuron-macrophage interaction is required to generate regeneration-associated BMDMs. ONCM concentration in MCM collected from BMDMs co-cultured with neurons accompanied by cAMP treatment was almost 10-fold higher than that in MCM from the co-cultures with PBS (Figure 2D). ONCM concentration was not significantly elevated in MCM obtained from macrophage only cultures. Next, we examined whether macrophage-derived ONCM is critical in this neuron-macrophage interaction model. When DRG neurons from ONCM deficient mice were co-cultured with WT macrophages, MCM from cAMP-treated conditions contained neurite outgrowth activity as robust as MCM from all WT cells (Figure 2E, G). ONCM concentration in this condition was comparable to that in MCM collected from the co-cultures consisting of WT neurons and macrophages (Figure 2H). When WT DRG neurons were co-cultured with ONCM-deficient macrophages, the neurite outgrowth activity in MCM from cAMP-treated conditions was markedly attenuated (Figure 2F-G). This decrease was associated with a marked decrease in ONCM concentration (Figure 2H), demonstrating that ONCM derived from RAMs plays an essential role in the robust neurite outgrowth activity in the *in vitro* neuron-macrophage interaction model.

### CCL2/CCR2 chemokine signaling regulates ONCM expression in macrophages

**Figure 3:**
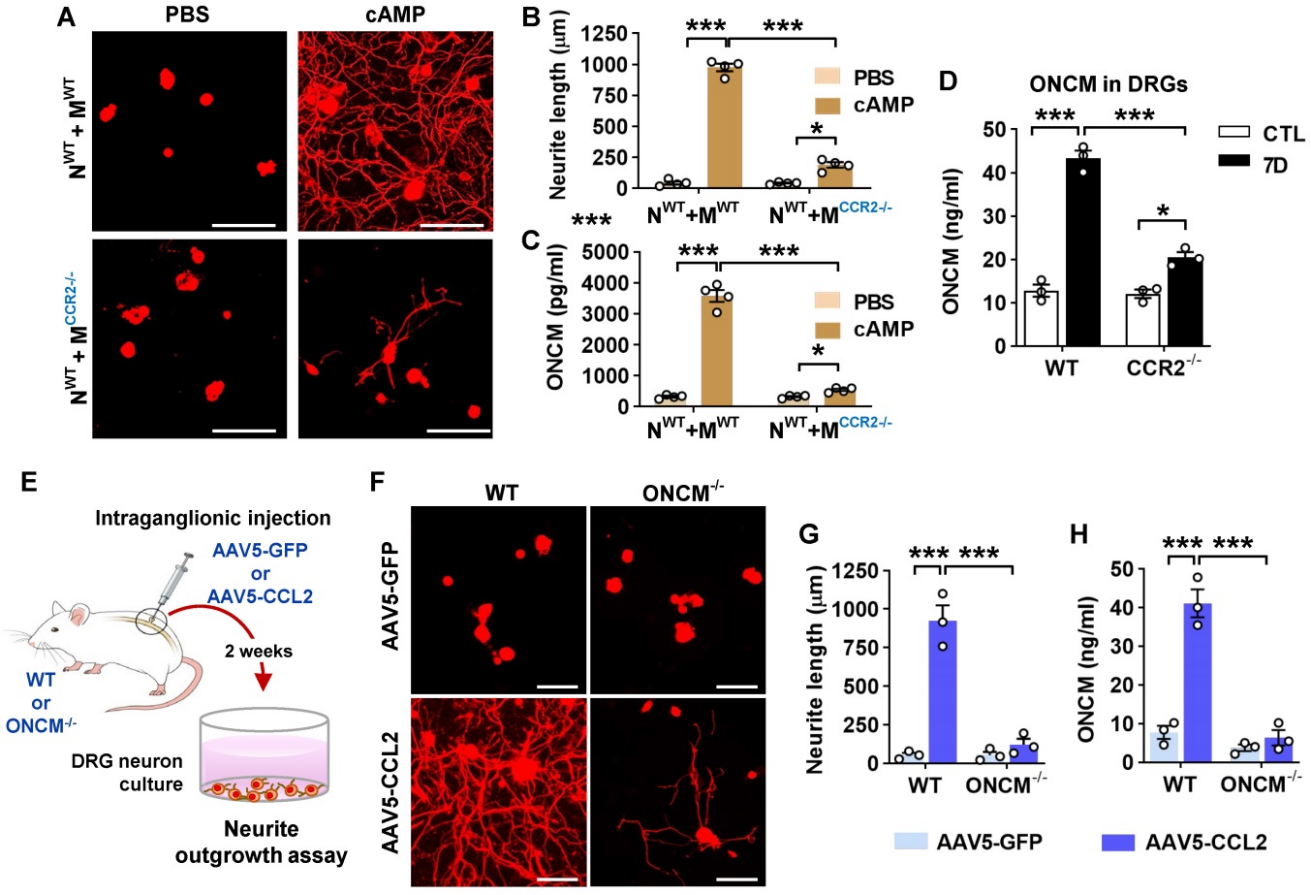
** CCL2/CCR2 chemokine signaling regulates ONCM expression in macrophages. A,** Representative images of neurite outgrowth in DRG neuron cultures treated with macrophage conditioned medium (MCM) obtained from neuron-macrophage cocultures using WT or CCR2-deficient (CCR2^-/-^) neurons (N) or macrophages (M). All cocultures were treated with either PBS or db-cAMP (cAMP), and the MCMs were collected for 72 h. DRG neurons and their neurites were visualized by immunofluorescence staining for beta3 tubulin. Scale bars represent 100 µm. **B,** Quantification graphs of neurite outgrowth in the presence of MCM from cultures of the different neuron-macrophage genotype combinations treated with PBS or cAMP. N = 4 independent cultures using independent MCMs for each condition. ***p < 0.001 and *p < 0.05 by unpaired *t* test. **C,** ELISA measurement of the ONCM concentration in the cell culture media obtained from different neuron-macrophage genotype combinations treated with PBS or cAMP. N = 4 independent cultures using independent MCMs for each condition. ***p < 0.001 and *p < 0.05 by unpaired *t* test. **D,** ELISA measurement of ONCM levels in DRGs from WT and CCR2^-/-^ mice 0 (CTL) and 7 d after sciatic nerve injury. *n* = 4 animals for each group. ***p < 0.001 and *p < 0.05 by unpaired *t* test. **E,** Experimental schematic diagram depicting the experimental processes. **F,** Representative images of neurons cultured from L5 DRGs freshly dissected from WT or ONCM^-/-^ mice subjected 2 weeks previously to intraganglionic injection of AAV5-GFP or AAV5-CCL2. The culture period was 15 h and DRG neurons and their neurites were visualized by immunofluorescence staining for beta3 tubulin. Scale bars represent 100µm. **G, H,** Quantification graphs comparing the mean neurite length (G) and the protein level of ONCM (H). N = 3 animals for each group. ***p < 0.001 and **p < 0.01 by unpaired *t* test.

We have previously shown that in the neuron-macrophage interaction model, CCR2-deficient peritoneal macrophages failed to produce pro-regenerative MCM 21. We confirmed that MCM obtained from CCR2-deficient BMDMs co-cultured with WT neurons and treated with cAMP showed markedly attenuated neurite outgrowth activity compared to that from WT BMDMs (Figure 3A-B). The reduction of neurite outgrowth activity was accompanied by low levels of ONCM protein in MCM from CCR2-deficient mice (Figure 3C). To determine if ONCM production *in vivo* is influenced by CCL2/CCR2 signaling, we performed preconditioning SNI in CCR2-deficient mice and measured the level of ONCM in DRGs. The level of ONCM concentration in DRG tissue increased at 7 dpi regardless of genotype (Figure 3D). However, the extent of SNI-induced increase in the level of ONCM was significantly smaller in CCR2-deficient mice than that in WT animals.

We have previously shown that intraganglionic injection of AAV5-CCL2 induced perineuronal macrophage activation and mimicked conditioning effects on neurite outgrowth in rats 21. We performed intraganglionic injection of AAV5-CCL2 into L5 lumbar DRGs in WT or ONCM-deficient mice and freshly cultured these DRG neurons 2 weeks after the injection (Figure 3E). Consistent with the previous study, intraganglionic AAV5-CCL2 injection resulted in a marked enhancement of neurite outgrowth in WT mouse DRG neurons (Figure 3F, G). This increase in neurite outgrowth was accompanied by an elevation of ONCM levels in DRGs (Figure 3H). However, the effects of CCL2 overexpression on neurite outgrowth were largely attenuated in ONCM-deficient mice (Figure 3F-G), and the level of ONCM concentration was not elevated by CCL2 overexpression in ONCM-deficient mice (Figure 3H). The number of perineuronal Iba-1 positive macrophages and the intensity of Iba-1 immunoreactivity in DRGs following AAV5-CCL2 injection were comparable between WT and ONCM-deficient mice (Figure S4A-B), corroborating that macrophage activation by CCL2/CCR2 signaling is not dependent on ONCM.

### ONCM potently increases neurite outgrowth in cultured DRG neurons by upregulating a distinctive set of RAGs

**Figure 4:**
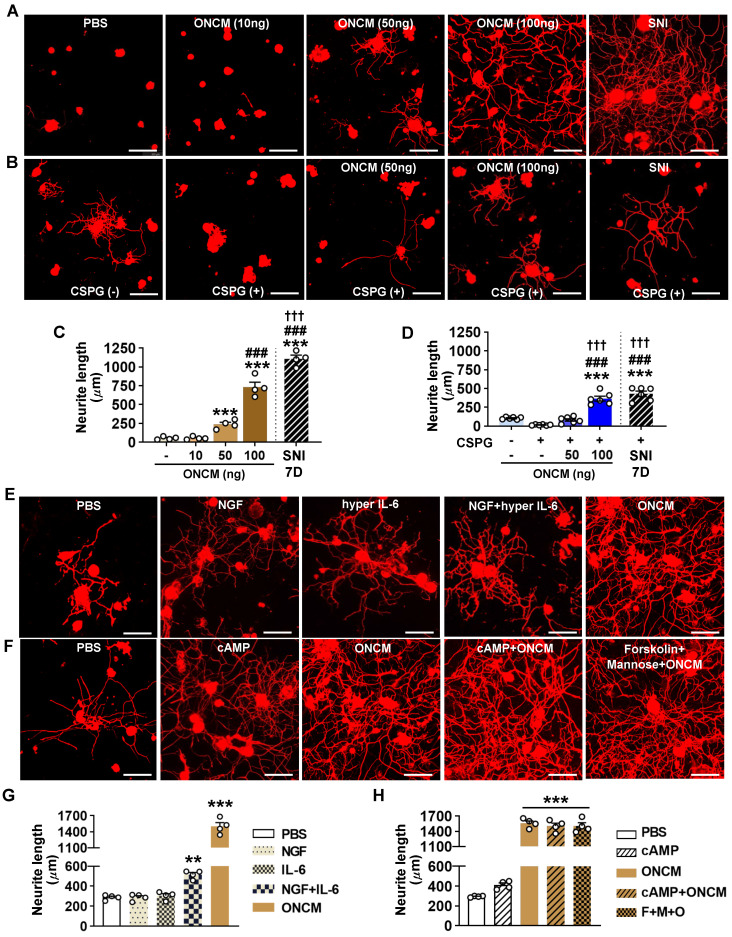
** ONCM potently increases neurite outgrowth in cultured DRG neurons. A,** Representative images of beta3 tubulin-positive cultured DRG neurons. Adult DRG neurons were cultured for 15 h with PBS or recombinant oncomodulin (ONCM) at 10, 50, and 100 ng concentrations. DRGs were freshly dissected from animals at 7 d after sciatic nerve injury (SNI) as positive control. Scale bars, 100µm. **B,** Representative images of neurite outgrowth of DRG neurons grown on a substrate precoated with chondroitin sulfate proteoglycans (CSPGs) for 24 h in response to different dosages of ONCM. DRG neurons dissected from animals at 7 d after SNI as positive control. At the 15 h culture period, neurite length was not appropriate to compare between the groups. Scale bars represent 100 µm. **C,** Quantification of the neurite outgrowth assay with ONCM treatment. N = 4 independent cultures for each condition. *** *p* < 0.001 and * *p* < 0.05 compared with PBS and 10 ng ONCM groups, ### *p* < 0.001 compared with 50 ng ONCM group, and ††† *p* < 0.001 compared with 100 ng ONCM treatment group by one-way ANOVA followed by Tukey's *post hoc* analysis. **D,** Quantification graph of the neurite outgrowth on CSPG coated substrate. pre-coated CSPG on neurite outgrowth assay with recombinant ONCM treatment. N = 5 independent cultures for each condition. ***p < 0.001 compared with CTL value, ###*p* < 0.001 compared with CSPG alone group, and ¶¶¶* p* < 0.001 compared with CSPG with 50 ng ONCM treatment group by one-way ANOVA followed by Tukey's *post hoc* analysis. **E,** Representative images of beta3 tubulin-positive cultured DRG neurons grown for 24 h with PBS, NGF, hyper IL-6, hyper IL-6 with NGF priming, and ONCM treatments. Scale bars represent 100 µm. DRG neurons were cultured for 24 h in this experiment to compare the extent of neurite outgrowth with the published data based on 24 h culture experiment. **F,** Representative images of neurite outgrowth in DRG neuron cultures treated with PBS, cAMP, ONCM, combination of cAMP and ONCM, and combination of mannose, forskolin, and ONCM. Scale bars represent 100 µm. DRG neurons were cultured for 24 h in this experiment to compare the extent of neurite outgrowth with the published data based on 24 h culture experiment. **G, H,** Quantification graphs of neurite outgrowth of cultured DRG neurons. N = 4 independent cultures for each condition. ***p < 0.001 and **p < 0.01 by one-way ANOVA followed by Tukey's *post hoc* analysis.

The finding that ONCM is required for the conditioning effects prompted us to test whether a direct application of recombinant ONCM protein to cultured DRG neurons can increase neurite outgrowth. ONCM treatment promoted neurite outgrowth in a dose-dependent manner, with the extent of neurite outgrowth by 100 ng ONCM to be approximately 70% of that achieved by preconditioning SNI (Figure 4A, C). Treatment of ONCM also overcame the growth inhibitory influence of CSPGs as coated substrate in a dose-dependent manner (Figure 4B), and 100 ng ONCM promoted neurite outgrowth as robustly as SNI (Figure 4D). Thus, ONCM possesses robust neurite outgrowth activity for cultured DRG sensory neurons. We compared the neurite outgrowth activity of ONCM with that of molecules with reported neurite outgrowth activity such as nerve growth factor (NGF) and IL-6 28, 29. Treatment of NGF at a dosage of either 10 or 100 ng did not enhance neurite outgrowth (Figure 4E, G). Hyper IL-6, a fusion protein of IL-6 and soluble IL-6 receptor facilitating intracellular transduction of IL-6 30, was also not effective. When hyper IL-6 was treated to DRG neurons primed with NGF, the extent of neurite outgrowth was significantly increased (Figure 4E, G). However, the neurite outgrowth activity of ONCM was still more robust than that of hyper IL-6 for NGF-primed neurons. It was reported that ONCM is effective in promoting optic nerve regeneration when combined with cAMP 23. ONCM increased neurite extension of retinal ganglion neurons *in vitro* when treated together with mannose and forskolin, a stimulator of adenylate cyclase, 23. In our experiment with DRG sensory neurons, ONCM alone exhibited very strong neurite outgrowth activity, and the combination of cAMP or mannose plus forskolin did not produce any significant additive effects (Figure 4F, H). To examine whether ONCM possesses neurite outgrowth activity in neural cells other than DRG sensory neurons, we treated cultured cortical neurons or spinal cord-derived neural stem cells (NSCs) with ONCM and measured the extent of neurite growth. We performed the cortical axon regeneration assay where cultured cortical neurons were scraped and allowed to regenerate their axons 31, 32. Treatment of ONCM at either 100 or 1000 ng/ml did not increase the extent of regenerating cortical neurites compared to the control condition (Figure S5A-B). When ONCM was treated for 24 h to cultured NSCs that were differentiated into the neuronal lineage, the length of beta3 tubulin positive neurites was not significantly different from that in the control group (Figure S5C-D).

**Figure 5:**
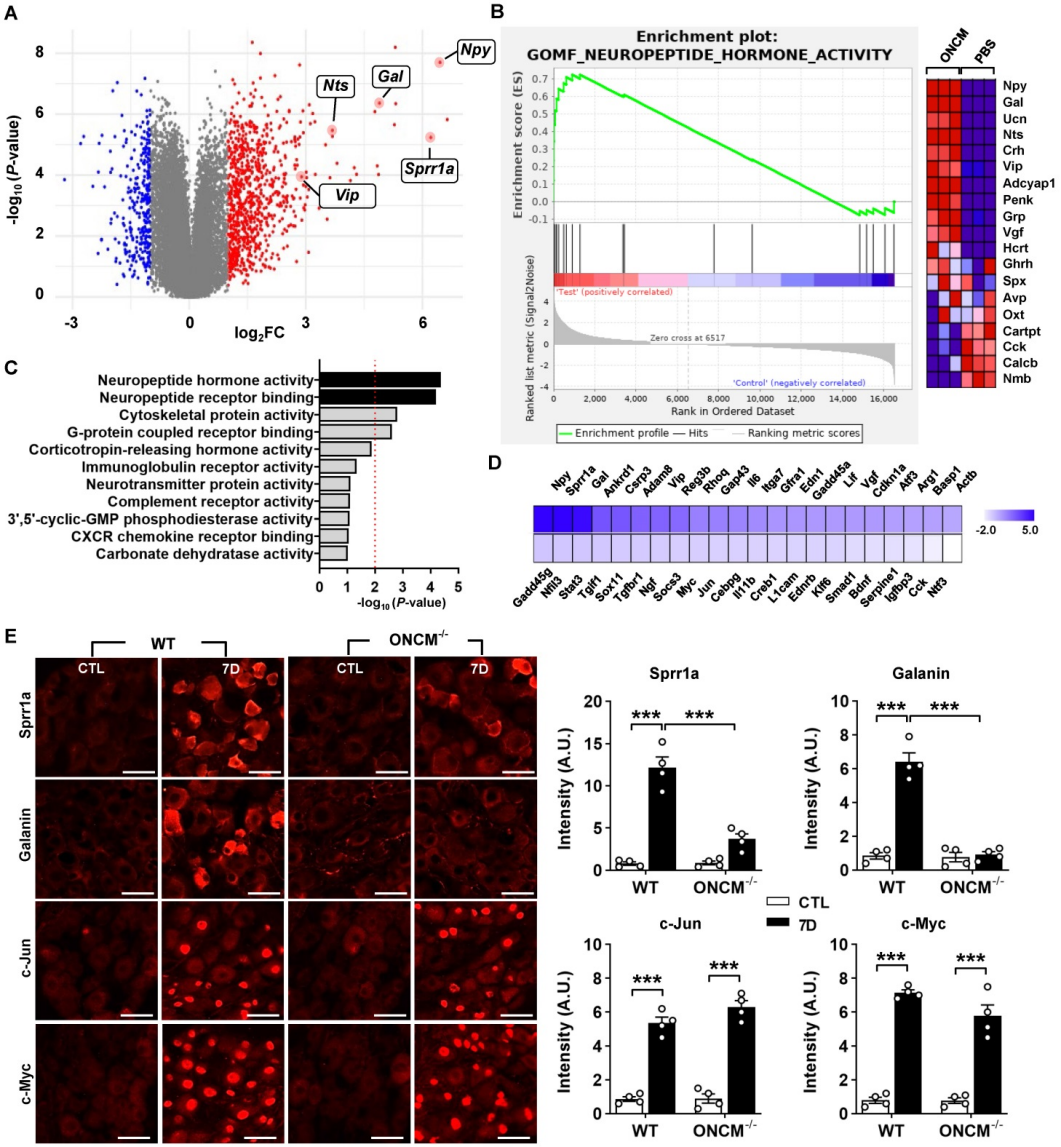
** ONCM upregulates a distinctive set of regeneration-associated genes. A,** Volcano plot of the gene expression profiles in DRG neurons treated with oncomodulin (ONCM). Blue and red dots indicate genes revealing a < 1.5-fold decrease and a > 1.5-fold increase in expression by ONCM, respectively. N = 3 biological replicates (independent cultures) for each group. **B,** Gene set enrichment analysis (GSEA) of ONCM-upregulated genes. The GSEA algorithm calculated an enrichment score reflecting the degree of over representation at the top or bottom of the ranked list of the genes included in a gene set in a ranked list of all genes present in the RNA-seq dataset. The analysis demonstrates that genes related to neuropeptide hormone activity are enriched in the ONCM treatment group. **C,** Gene ontology analysis of upregulation genes revealing a > 1.5-fold increase in expression by ONCM. Top 11 GO terms for molecular function ranked by fold enrichment were shown in the bar graph. Black bars indicate neuropeptide-related GO term. **E,** Color-coded heatmap of the gene expression levels for selected 44 RAGs. F, Representative images of Sprr1a, Galanin, c-Jun, and c-Myc immunoreactivity in DRGSs of WT and ONCM^-/-^ mice at 0 (CTL) and 7 d after SNI. Quantification of intensity of immunoreactivities in WT and ONCM^-/-^ mice 0 (CTL) and 7 d after SNI. N = 4 animals for each condition. Scale bars represent 50 µm. ***p < 0.001 by one-way ANOVA followed by Tukey's *post hoc* analysis.

To gain insight into potential mechanisms downstream of ONCM for this potent neurite outgrowth activity, we performed RNA-seq using cultured DRG neurons treated with ONCM for 12 h. Since the method of culturing DRG neurons cannot completely remove non-neuronal cells, we enriched neurons using bovine serum albumin (BSA) cushion 33. BSA cushion effectively diminished expression of several non-neural cell-specific genes (Figure S6). Transcriptomic analysis revealed that 2535 and 1973 genes were upregulated and downregulated, respectively, by more than 1.5 folds (Figure 5A). It was notable that several neuropeptide family genes, such as *Npy* (Neuropeptide Y), *Gal* (Galanin), and *Vip* (Vasoactive intestinal peptide), were highly upregulated. Gene set enrichment analysis (GSEA) showed that the gene set related to neuropeptide hormone activity was highly enriched in the upregulated genes in response to ONCM treatment (Figure 5B). GO analysis also revealed that neuropeptide signaling terms were enriched (Figure 5C). Upregulation of the neuropeptide genes was validated in independent DRG neuron samples (Figure S7A). Furthermore, we found that expression of *Npy* and *Gal*, along with *Sprr1A,* which is a well-characterized RAG and highly upregulated in the transcriptomic analysis, were highly induced in DRGs following SNI (Figure S7B). The increases of those genes at 7 dpi were markedly attenuated in ONCM-deficient mice.

Preconditioning SNI elicits upregulation of a group of genes that are collectively called “regeneration-associated genes (RAGs)”^9^, ^34^. We previously selected a set of 44 RAGs that had been consistently reported as upregulated genes in previously published gene expression studies in DRGs 35. To determine the potential contribution of ONCM to the SNI-induced RAG profile, we analyzed the expression of the 44 RAGs in ONCM-treated cultured DRGs. Most of the neuropeptide-related genes upregulated by ONCM (such as *Npy*, *Gal*, and *Vip*) belonged to the selected RAG set (Figure 5D). *Sprr1a* and *Gap43*, which are representative genes reflecting the intrinsic capacity of axon growth, were significantly affected by ONCM. ONCM also significantly increased the expression of *Ankrd1*, *Csrp3* (also known as *Mlp*), and *Reg3b*, of which neurite outgrowth activity was experimentally demonstrated 36-38. RAGs related to ECM and cell adhesion (*Adam8* and *Itga7*) and cytokine/growth factors (*Il6*, *En1*, *Gfra1*) were also significantly, but to a lesser extent, increased in response to ONCM. In contrast, the expression of transcription factor RAGs such as *Stat3*, *Myc*, *Jun*, and *Smad2*, which are known to play a role of hub transcription factors to regulate modular expression of RAG subsets 9, 35, was not significantly changed (Figure 5D). To ascertain whether certain RAGs are independent of ONCM, we compared the protein expression of RAGs following SNI between WT and ONCM-deficient mice. As expected, immunoreactivities against Galanin and Sprr1A were dramatically induced at 7 days following SNI and noticeably attenuated in ONCM-deficient mice (Figure 5E-F). However, the injury-induced nuclear immunoreactivities to c-Jun and c-Myc transcription factors were not reduced in ONCM-deficient mice (Figure 5E-F).

### Overexpression of ONCM construct with the signal sequence in DRGs enhances axon growth capacity

**Figure 6:**
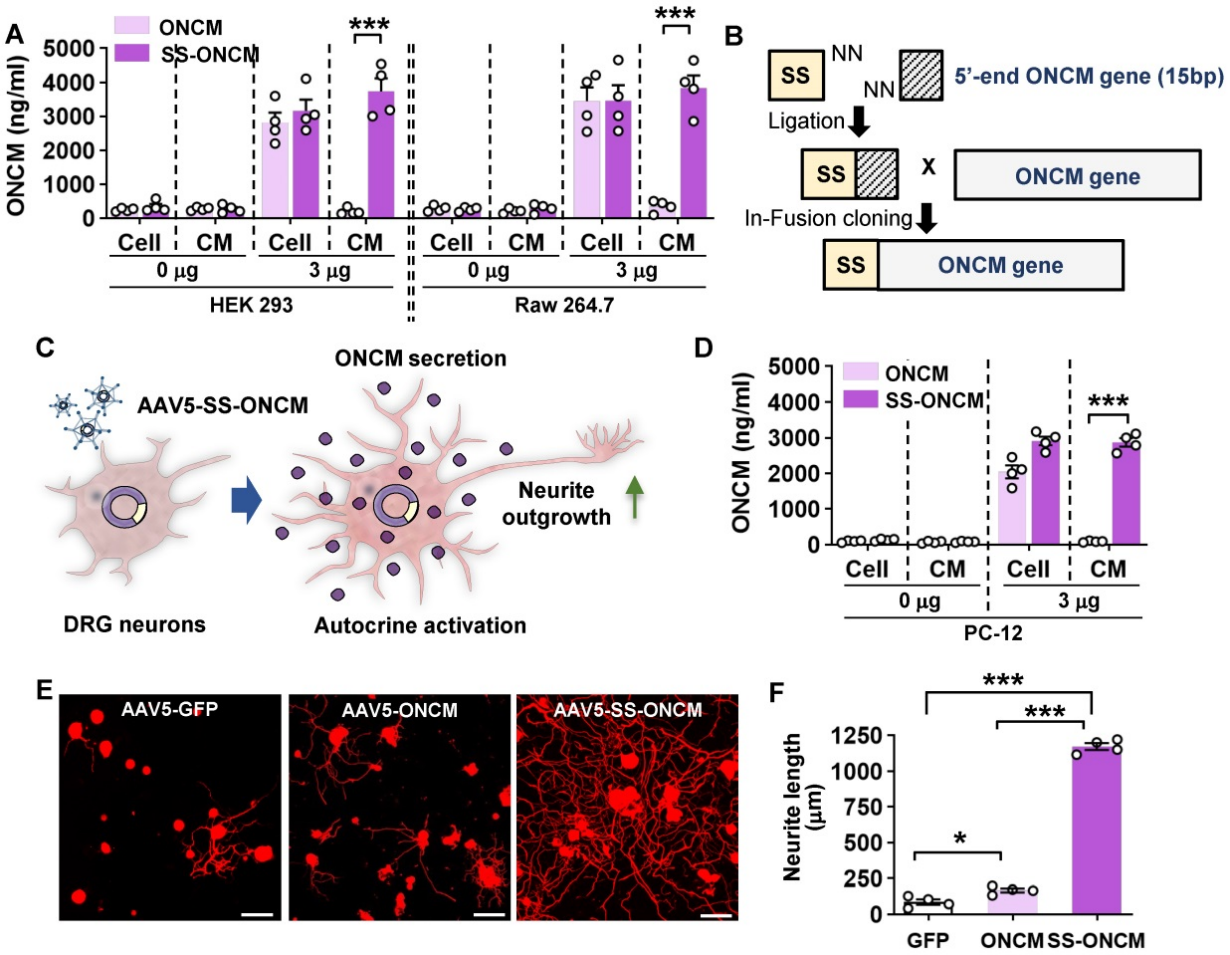
** Overexpression of oncomodulin construct with the signal sequence in DRGs enhances axon growth capacity. A,** ELISA results for the oncomodulin (ONCM) level in the cell lysates and the culture medium from cultured HEK 293 and Raw 264.7 cells transfected with ONCM with or without the signal sequence (SS). N = 4 independent cultures for each condition. ***p < 0.001 by unpaired *t* test. **B,** Schematic representation of a construct with the signal sequence (SS) for secretion of ONCM. **C,** Schematic diagram of AAV-mediated transfection with ONCM construct containing the signal sequence (AAV5-SS-ONCM) in the DRG neurons and autocrine activation of neurite outgrowth. **D,** ELISA measurement of the ONCM concentration in the cell lysate and the conditioned medium from cultured PC 12cells transfected with ONCM with or without the signal sequence (SS). N = 4 independent cultures for each condition. ***p < 0.001 by unpaired *t* test. E, Representative images of neurons cultured from L5 DRGs freshly dissected from animals subjected to intraganglionic injection of AAV5-GFP, AAV5-ONCM or AAV5-SS-ONCM 2 weeks prior to culture. The culture period was 15 h and DRG neurons and their neurites were visualized by immunofluorescence staining for beta3 tubulin. Scale bars represent 100 µm. **F,** Quantification graph comparing the mean neurite length. ***p < 0.001 and *p < 0.01 by one-way ANOVA followed by Tukey's *post hoc* analysis.

We sought to overexpress ONCM in DRGs to examine whether ONCM is sufficient to enhance the capacity of axon regeneration in DRG sensory neurons. When ONCM plasmids were transfected into HEK 293 cells, ONCM expression was detected in cell lysates but not in the culture medium (Figure 6A). ONCM cDNA was also delivered to Raw 264.7 mouse macrophage cells, but still the level of ONCM secreted to the media was negligible (Figure 6A), suggesting that exogenously delivered ONCM gene could not utilize the secretory machinery in macrophages. We found that the ONCM gene is lacking the signal sequence at its N-terminus, which encodes the signal peptide that directs newly synthetized proteins toward the secretory pathway. To facilitate extracellular secretion of ONCM, DNA sequences encoding bacterial alkaline phosphatase signal peptide were inserted into the ONCM cDNA construct (Figure 6B). Transfection of the ONCM construct with the signal sequence (SS-ONCM) increased the amount of ONCM in the culture medium from HEK 293 and mouse macrophage cells (Figure 6A). Since virus-mediated gene delivery to macrophages *in vivo* is challenging 39, we conceived an alternative approach of AAV-mediated transfection of DRG neurons with ONCM construct containing the signal sequence, delivering ONCM to the neurons in an autocrine manner (Figure 6C). We confirmed that adjoining the signal sequence facilitated ONCM secretion in PC12 neuronal cells as well (Figure 6D). Intraganglionic injection of AAV5-ONCM resulted in a slight but significant increase in neurite length when the DRGs were acutely cultured (Figure 6E-F). In comparison, AAV5-SS-ONCM containing the signal sequence dramatically increased the extent of neurite outgrowth by almost 10 folds, supporting the notion that extracellularly secreted ONCM is sufficient to increase the capacity of axon growth in DRG sensory neurons.

### Encapsulation via a nanogel drug delivery system sustains stability of ONCM activity

**Figure 7:**
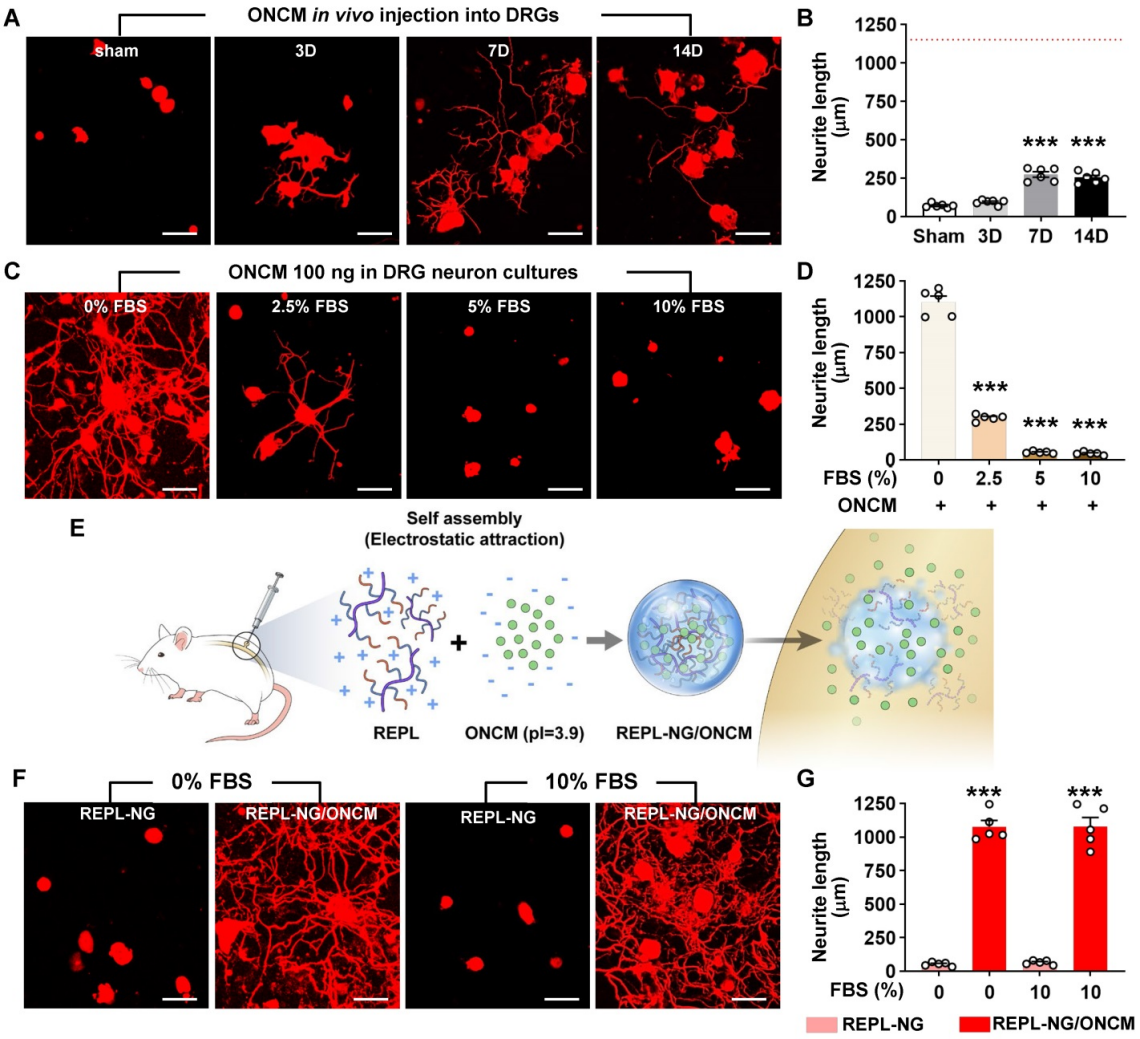
** Encapsulation via a nanogel drug delivery system sustains stability of ONCM activity. A, B,** Comparison of the mean neurite length between cultures from intraganglionic injection of ONCM 0 (sham), 3, 7, and 14 d after injection. Scale bars, 100 µm. N = 6 animals per group. ***p < 0.001 compared with control values by one-way ANOVA followed by Tukey's *post hoc* analysis. The red dotted line indicates the average value of the neurite length of DRG neurons taken from animals with preconditioning injury. **C,** Adult DRG neurons were cultured for 15 h being treated with 100 ng ONCM. Fetal bovine serum (FBS) was added to the culture at 0, 2.5, 5, and 10% concentrations. Scale bars, 100 µm. **D,** Quantification of the DRG neurite outgrowth assay. N = 5 independent cultures for each condition. ***p < 0.001 compared with 0% FBS with ONCM value by one-way ANOVA followed by Tukey's *post hoc* analysis. **E,** A simplified illustration of the *in vivo* ONCM delivery system by nanogel-based drug delivery system, reducible ɛ-poly(_L_-lysine) (EPL)-based nanogel (REPL-NG). **F,** Representative images of neurite outgrowth of cultured DRGs without FBS or 10% FBS treated with REPL-NG or REPL-NG/ONCM. The culture duration was 15 h. Scale bars, 100 µm. **G,** Quantification of the DRG neurite outgrowth assay. N = 5 independent cultures for each condition. ***p < 0.001 compared with REPL-NG groups by one-way ANOVA followed by Tukey's *post hoc* analysis.

To develop an ONCM-based therapeutic strategy that is more amenable to clinical translation, we attempted to deliver recombinant ONCM protein to DRGs. We tested whether recombinant ONCM injection to DRG neurons could enhance the extent of neurite outgrowth from DRG neurons that were acutely cultured at 3, 7, and 14 days after injection. Injection of the recombinant ONCM protein increased the mean neurite length at 7 and 14 days, but the extent of neurite outgrowth was much less than what is expected from preconditioning SNI (Figure 7A-B). The apparent difference between the ONCM effects* in vitro* and *in vivo* led us to consider an issue of ONCM protein stability when delivered to the DRG tissue *in vivo*. To test if the biological activity of ONCM would be affected by *in vivo* tissue environment, we added fetal bovine serum (FBS) at different concentrations to the culture medium where DRG neurons were cultivated with ONCM. Surprisingly, the addition of the serum attenuated the neurite outgrowth activity of ONCM in a dose-dependent manner (Figure 7C-D), suggesting that ONCM activity would be strongly inhibited by tissue fluids in an *in vivo* environment.

To improve the stability of ONCM protein *in vivo*, we developed a novel nanogel-based drug delivery system, reducible ɛ-poly(_L_-lysine) (EPL)-based nanogel (REPL-NG). Positive charges of the primary amines in REPL-NG could encapsulate ONCM, which has a strong negative charge (pI = 3.9) in physiological pH (i.e., pH 7.4), via an electrostatic force and thereby increase its *in vivo* stability (Figure 7E). When nanogel-encapsulated ONCM was treated instead of free recombinant ONCM, neurite outgrowth was robustly enhanced regardless of adding 10% serum (Figure 7F), demonstrating that REPL-NG/ONCM complex successfully protected ONCM activity from the serum components.

**Figure 8:**
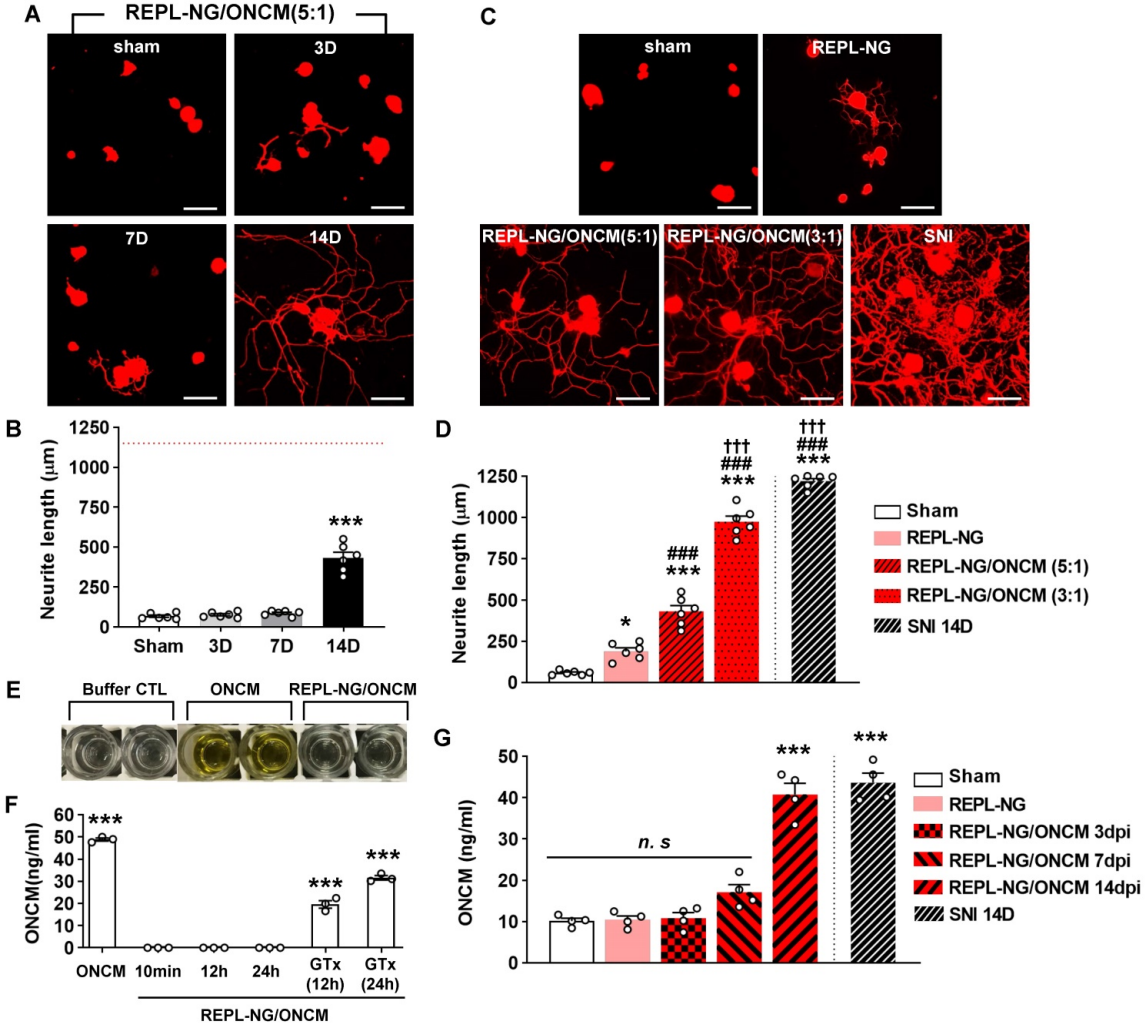
** REPL-NG/ONCM complex enables sustained activity of ONCM *in vivo*. A,** Representative images of neurite outgrowth of DRG neurons taken from animals with intraganglionic injection of REPL-NG with ONCM complexed at 5:1 ratio, REPL-NG/ONCM(5:1) at 0 (sham), 3, 7 and 14 d after injection. Neurons from the L4 and L5 DRGs were cultured for 15 h before being fixed for the immunofluorescent visualization of neurites with anti-beta3 tubulin. Scale bar, 100 µm. **B,** Comparison of the mean neurite length between cultures. N = 6 animals per group. ***p < 0.001 compared with control values by one-way ANOVA followed by Tukey's *post hoc* analysis. The red dotted line indicates the average value of the neurite length of DRG neurons taken from animals with preconditioning sciatic nerve injury (SNI). **C,** Neurite outgrowth of cultured DRGs neurons taken from animals with intraganglionic injection of vehicle (sham), REPL-NG, REPL-NG/ONCM(5:1), and REPL-NG/ONCM(3:1) at 14 d after injection or animals subjected to preconditioning SNI 14 days ago. Neurons from the L4 and L5 DRGs were cultured for 15 h before being fixed for the immunofluorescent visualization of neurites with anti-beta3 tubulin. Scale bar, 100 µm. **D,** Quantification graph comparing the mean neurite length. ***p < 0.001 and *p < 0.01 compared with sham group; ###* p* < 0.001 compared with REPL-NG group; †††*p* < 0.001 compared with REPL-NG/ONCM(5:1) group by one-way ANOVA followed by Tukey's *post hoc* analysis. **E,** Microtiter plates showing the colorimetric reaction of ONCM ELISA assay. Wells indicate lysis buffer only (buffer CTL), ONCM freely dissolved (ONCM), and REPL-NG/ONCM in lysis buffer 10 min after incubation. It was notable that there was almost no color change in wells with REPL-NG/ONCM as compared with those containing ONCM. **F,** ELISA measurement of ONCM levels in the lysis buffer containing detergent with free recombinant ONCM or nanogel-encapsulated ONCM (REPL-NG/ONCM). Incubating REPL-NG/ONCM in the lysis buffer for up to 24 h did not result in a release of ONCM detectable by ELISA. Glutathione (GTx) was added at 10 mM concentration for 12 or 24 h to induce bursting of the nanocomplex. N = 3 independent experiments for each group. ***p < 0.001 compared with control group by one-way ANOVA followed by Tukey's *post hoc* analysis. **G,** ELISA measurement of ONCM concentration in the DRGs obtained at 3, 7, and 14 days after intraganglionic injection of REPL-NG/ONCM. N = 4 animals per each group. ***p < 0.001 compared with control group by one-way ANOVA followed by Tukey's *post hoc* analysis.

The REPL-NG/ONCM complex was injected into the DRGs to examine the extent of neurite outgrowth from acutely cultured DRGs. REPL-NG with ONCM complexed at a 5:1 ratio, REPL-NG/ONCM(5:1), significantly enhanced axon growth of DRG neurons compared to no injection control at 14 days after injection (Figure 8A). However, the extent of neurite growth was still smaller than that achieved by preconditioning SNI (Figure 8A-B). Then, we increased the composition of ONCM to generate REPL-NG/ONCM(3:1) that can deliver a higher amount of ONCM (1.7 µg for 3:1 vs. 1.0 µg for 5:1). We also anticipated that the relative decrease of the REPL-NG in the new complex at 5:1 ratio would reduce the electrostatic interaction leading to facilitation of free ONCM release from the NG complex. Injection of the REPL-NG/ONCM(3:1) increased neurite outgrowth at 14 dpi more markedly than the REPL-NG/ONCM (5:1), to an extent comparable to that achieved by preconditioning SNI (Figure 8C-D). Injection of REPL-NG without ONCM did not significantly affect neurite outgrowth. Encapsulation of ONCM by REPL-NG is supposed to result in a slow release of free ONCM. To test this notion *in vivo*, we tried to measure the level of released ONCM in DRG tissue following injection of the nanocomplex. We first confirmed that the ELISA assay cannot detect encapsulated ONCM (REPL-NG/ONCM) even though it was exposed to tissue lysis buffer containing detergent (Figure 8E-F). Incubating the nanocomplex in the lysis buffer for up to 24 h did not result in a release of free ONCM measurable by ELISA (Figure 8F). REPL-NG possesses multiple disulfide bonds that can be reduced by intracellular glutathione facilitating biodegradation within a cellular environment. When glutathione was treated for longer than 12 h to the solution containing encapsulated ONCM, ONCM was detectable by ELISA (Figure 8F), indicating that only ONCM that was released from the nanogel complex can be measured by ELISA assay. We found that the amount of ONCM in DRGs increased only slightly at 3 and 7 dpi, but very markedly at 14 dpi (Figure 8G), demonstrating that free ONCM is slowly released from the nanogel complex enabling sustained activity of ONCM *in vivo*. Injection of REPL-NG/ONCM did not invoke strong inflammatory responses in DRGs, and there was no evidence of significant damage to DRG neurons by the nanocomplex (Figure S8).

### Nanogel-mediated delivery of ONCM protein to DRGs achieves robust central regeneration of dorsal column axons beyond spinal cord lesion

**Figure 9:**
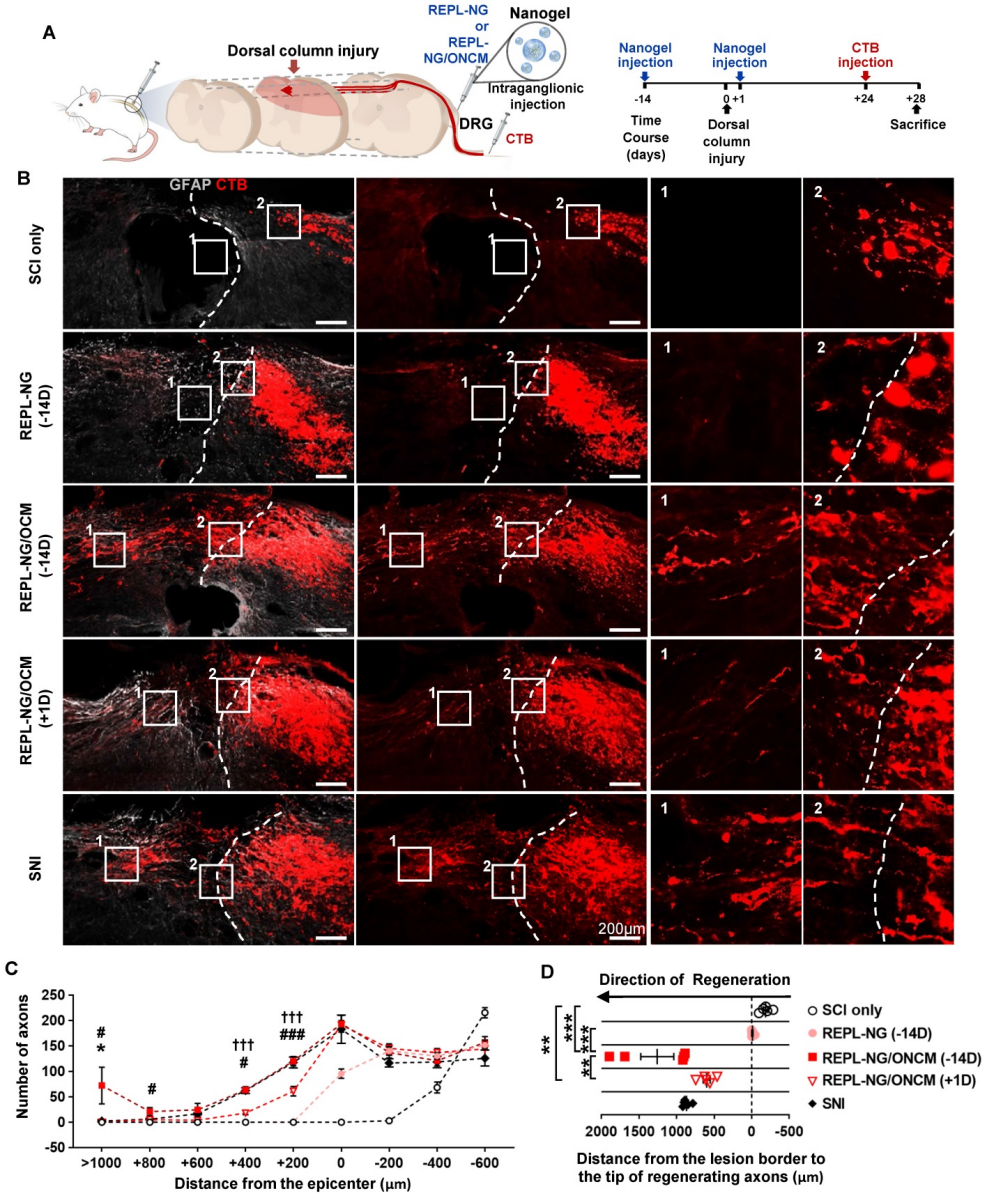
** REPL-NG/ONCM complex robustly increases regeneration of dorsal column axons following spinal cord injury. A,** Experimental scheme depicting the time course of the experimental procedures. **B,** Representative images of cholera toxin B (CTB)-labeled axons (red) and GFAP-immunostained spinal cord sections (gray) from animals subjected to spinal cord dorsal hemisection only or injected with REPL-NG 14 d before injury (REPL-NG; -14D), with REPL-NG/ONCM 14 d before injury (REPL-NG/ONCM; -14 D), or with REPL-NG/ONCM 1 d after injury (REPL-NG/ONCM; +1D), and those subjected to preconditioning sciatic nerve injury (SNI) 2 weeks before creating the spinal lesion. Dashed lines indicate caudal lesion borders as determined by GFAP immunostaining. The boxed regions are magnified in the right panels. Scale bars represent 200 µm. **C,** Quantification graph comparing the number of CTB-positive axons within counting blocks located at different distances from the lesion border. The numbers in the x axis indicate the distance of the rostral border of a counting block from the lesion border. *p < 0.01 compared between SNI and REPL-NG/ONCM (-14D) groups; ###* p* < 0.001 and #*p* < 0.01 compared between REPL-NG/ONCM (-14D) and REPL-NG/ONCM (+1D) values; †††*p* < 0.001 compared between SNI and REPL-NG/ONCM (+1D) groups by one-way ANOVA followed by Tukey's *post hoc* analysis. Statistical differences in reference to SCI only or REPL-NG groups were omitted for clarity. **D,** Quantification graph comparing the longest distance of regenerating axons from the caudal lesion border. Individual circles plot the longest distance of each animal. Negative values indicate the degree of the retraction from the lesion border. ***p < 0.001**p < 0.01; *p < 0.05; between indicated groups by one-way ANOVA followed by Tukey's *post hoc* analysis. N = 6 animals for each group.

We tested the effects of the nanogel-ONCM complex on central axon regeneration in an *in vivo* spinal cord injury model. We created dorsal hemisection at the T9 spinal cord to cut ascending dorsal column sensory axons in the spinal cord (Figure 9A). REPL-NG/ONCM(3:1) was injected into L4 and L5 DRGs 14 days before or 1 day after the spinal cord injury (SCI), and the extent of regeneration traced by cholera toxin B (CTB) was compared to animals with injury only, REPL-NG only, or subjected to preconditioning SNI. The dorsal hemisection resulted in retraction of CTB traced DRG sensory axons (Figure 9B). Injection of REPL-NG alone without ONCM conjugated slightly counteracted the axonal retraction, but there were virtually no axons growing beyond the caudal injury border. In contrast, REPL-NG/ONCM injection 2 weeks before the spinal lesion led to robust regeneration beyond the lesion border (Figure 9B-C). CTB traced axons traversing the lesion site exhibited a long-range linear growth along the rostrocaudal direction reaching up to 2 mm rostral to the caudal injury border (Figure 9B, D). The mean number of regenerating axons beyond the caudal lesion border was comparable to that achieved by preconditioning SNI up to 600 µm from the injury epicenter, but axons regenerating at the region more rostral to 600 µm were observed only in animals with REPL-NG/ONCM injection 14 days before the lesion (Figure 9C-D). To assess the feasibility of the REPL-NG/ONCM as a clinically applicable therapeutic option, we injected REPL-NG/ONCM one day after injury. Although post-injury injection also resulted in enhanced axon regeneration, the extent was considerably decreased compared to the pre-injury injection (Figure 9B-D). The central axon regeneration by REPL-NG/ONCM was accompanied by strong expression of a neuropeptide-related gene, Galanin, in DRGs (Figure S9).

## Discussion

There is mounting evidence that non-neuronal cells in DRGs may contribute to enhanced axon growth potential by a well-characterized “preconditioning injury” paradigm 12, 14, 18, 40. Among various non-neuronal cells, the functional significance of macrophage involvement was thoroughly documented by either pharmacological inhibition using minocycline 16 or genetic ablation of CCR2 17. Perineuronal macrophage activation in DRGs following peripheral nerve injury is also implicated in the evolution of neuropathic pain 41-43. In CNS, microglial activation surrounding axotomized neurons was linked to axonal regeneration or changes in synaptic structures 44, 45. These findings strongly suggest that perineuronal myeloid cells exert a profound influence on neurons of which axons are injured at a distant site. Previous studies have identified various signals that DRG sensory neurons transmit following axonal injury to activate macrophages 21, 46, 47. However, it remains largely unknown how exactly the perineuronal macrophages affect neurons in return to support their potential of regenerating injured axons.

The present study provides several lines of evidence that ONCM is the macrophage-derived effector signal to enhance the intrinsic capacity of DRG sensory neurons for axonal regeneration. First, ONCM expression was increased exclusively in DRG macrophages following SNI in a manner dependent on CCR2, a receptor for CCL2 ligand that was shown to recruit and activate macrophages 21, 47, 48. Second, SNI did not lead to enhancing axon growth capacity in ONCM-deficient mice when DRG neurons were cultured at 7 dpi, demonstrating the necessity of ONCM. However, ONCM was not required for early induction of conditioning effects at 2 dpi, a time point when an injury-induced increase in macrophages has not yet taken place 49. This finding suggests that the contribution of ONCM becomes significant only when macrophages are substantially activated. Third, ONCM-deficient macrophages failed to produce MCM with neurite outgrowth activity in the *in vitro* neuron-macrophage interaction model. In the same model, MCM from WT macrophages co-cultured with ONCM-deficient neurons contained potent neurite outgrowth activity, precluding the possibility that neuron-derived ONCM is involved in the injury-induced conditioning effects. Finally, overexpression of ONCM engineered to be released extracellularly in DRGs *in vivo* increased the axon growth capacity of DRG neurons, successfully mimicking the conditioning effects. An intriguing finding was the absence of the signal sequence within the ONCM coding region, although ONCM was readily released into MCM from cultured primary macrophages. It remains to be determined how ONCM is released from macrophages not utilizing the canonical secretory pathway.

We found that ONCM possesses highly robust neurite outgrowth activity *in vitro*, to the extent comparable to that achieved by preconditioning injury. When cultured DRG neurons were exposed to other molecules such as NGF and IL-6 (used as hyper IL-6 here), or IL-6 with NGF priming 28, 29, the extent of neurite outgrowth in response to ONCM was exceedingly higher. Our finding that ONCM successfully overcame the inhibitory influence of CSPGs was also in contrast with the previous report showing no effect of ONCM in *in vitro* spot gradient assay 50. These discrepancies between our and previous findings might be due to different culture conditions or a species difference between mouse (our study) and rat (the above previous studies) DRG neurons. Nonetheless, it could be argued that ONCM is the most potent molecular factor to induce neurite outgrowth in culture DRG neurons at least in our culture condition. ONCM was identified as a potent signal for axon regeneration of retinal ganglion cells (RGCs) 23. ONCM *in vitro* did not induce RGC axon outgrowth when treated alone, but potently increase the axon growth when combined with mannose and forskolin 23. In our study, ONCM treatment alone, without the combination of the above co-factors, achieved the almost maximum extent of axon growth that could be expected following preconditioning injury. We found that ONCM did not exert neurite outgrowth activity in cultured cortical neurons or NSCs, indicating that ONCM effects are highly specific for DRG sensory neurons as well as RGCs. This specificity would probably implicate tissue-specific expression of its receptor that remains to be identified. The precise mechanism by which ONCM potently increases neurite outgrowth in DRG neurons is presently unknown. Our RNA-seq data suggest that neuropeptide-related genes may be downstream effectors for ONCM effects on neurite outgrowth. Potential roles of the neuropeptide-related genes in sensory axon regeneration were demonstrated. For example, disruption of the *Gal* gene reduced the regenerative capacity of sensory neurons 51. Furthermore, intrathecal administration of NPY and VIP resulted in an increase in the percentage of dissociated DRG neurons with growing neurites 52. Interestingly, *Gal* gene was highly expressed in retinal ganglion cells following axotomy plus lens injury in which retinal ganglion cell axon regeneration is promoted via macrophage derived ONCM 53. In contrast, ONCM treatment did not significantly increase the expression of hub transcription factors implicated in the conditioning effects. We verified persistent activation of c-Jun and c-Myc following SNI in ONCM-deficient mice, indicating that ONCM is a downstream effector molecule of which expression may be influenced by those hub transcription factors following preconditioning injury.

To facilitate the effective delivery of ONCM protein to DRGs, the REPL-NG drug delivery system was adopted in this study. NGs are nano-sized (usually 100-200 nm) particles with physical properties of hydrogels formed by crosslinked swellable polymer networks 54, 55. Owing to its capacity to load a large amount of drugs and the fluid-like deformability 54, 56, we speculated that NG would be an ideal carrier targeting organs of very small size like the DRG. Furthermore, NG can protect biomolecular cargos from degradation or elimination and thereby enhance the stability of target molecules exposed to biological fluid 55. After thiolation and successive oxidation of EPL, the resulting REPL-NG possesses multiple disulfide bonds and primary amines. Primary amines exhibit positive charges when REPL-NG is dissolved in a solution with pH 7.4. Ionic interaction with charged polymers and counter-charged proteins has been employed for the controlled release of protein drugs 57, 58. The positive charge in REPL-NG is exploited to form an electrostatic interaction with negatively charged ONCM with a pI value of 3.9. This electrostatic force allows nano-sized complexation between REPL-NG and ONCM and would impart stability of ONCM *in vitro* with the serum components and *in vivo* DRG tissue. Indeed, this REPL-NG/ONCM complex completely hindered antibody binding to ONCM even in the presence of detergent-containing lysis buffer, resulting in no detection of ONCM in ELISA assay. Strong and stable encapsulation of ONCM was also supported by the observation that the ONCM level in DRGs was slowly increased by 14 days after being injected as REPL-NG/ONCM complex. REPL-NG/ONCM is supposed to be delivered within cells of interest as a form of nanocomplex. Glutathione-triggered cleavage of disulfide bonds is often utilized for intracellular drug delivery in a controlled manner 59-61. Multiple disulfide bonds in REPL-NG can be degraded by intracellular glutathione releasing free ONCM within a cytoplasmic compartment. Our finding that the addition of glutathione burst REPL-NG/ONCM enabling detection of ONCM by ELISA supports the notion that intracellular glutathione would be a key mechanism for efficient intracellular delivery of ONCM. Collectively, our study demonstrated that encapsulation of ONCM by REPL-NG could greatly improve stability and prolong its bioactivity in DRG tissue.

We observed that NG-mediated delivery of ONCM promoted regeneration of sensory axons well beyond spinal cord lesion, to the extent surpassing that observed following preconditioning injury. The longest distance of regenerating axons from the lesion center was almost 2 mm, and this amount of regeneration would be certainly notable when compared to our own experiences with overexpression of a chemokine or a transcription factor in the exact same injury model 21, 35. We speculate that this remarkable level of axon regeneration was due to enhanced stability and sustained bioactivity of ONCM protein in DRG tissue. As exemplified by the inhibition of ONCM activity by serum components in this study, peptide drugs like growth factors known to stimulate axon regeneration would be susceptible to various molecular factors within the tissue environment and it would be challenging to maintain their bioactivity in a prolonged time scale. The stability issue would be even more pivotal in axon regeneration studies since cellular processes of axon regeneration require quite a long time scale with at least a couple of weeks in rodents. One caveat is that the stability of a peptide drug should be optimized for the best outcome. In this study, we found that injection of REPL-NG/ONCM one day after a spinal cord lesion did not lead to axon regeneration as robust as the same injection 2 weeks before the lesion. Based on the temporal course of ONCM level in DRGs following injection, it is conceivable that the availability of free ONCM may not be sufficient for the duration of initial one or two weeks following spinal cord lesion, when key biological mechanisms for axon regeneration should be fully mobilized to stimulate elongation of cytoskeletal proteins. Further studies will address this optimization issue to produce the best outcome by post-injury injection of REPL-NG/ONCM within a clinically relevant therapeutic window.

## Materials and methods

### Animals

Adult female Sprague Dawley rats (250-300 g) and C57BL/6 wild-type (WT) mice were purchased from Orient Bio Inc. ONCM- and CCR2-deficient mice on C57BL/6 background were purchased from the European Mouse Mutant cell repository (MGI ID 4431716) and The Jackson Laboratory (stock 004434), respectively. Cx3cr1-GFP mice on C57BL/6 background were purchased from Jackson Laboratory (stock 005582). The Institutional Animal Care and Use Committee of Ajou University School of Medicine approved all animal protocols.

### Surgical procedures

Mice were anesthetized with an intraperitoneal injection of the ketamine and xylazine mixture (100 mg/kg and 10mg/kg, respectively). For the creation of SNI, muscles were displaced to expose the right sciatic nerve, and the nerve was ligated proximal to its trifurcation. The sciatic nerve was completely transected below the ligation site with fine surgical scissors. Rats were anesthetized with an intraperitoneal injection of the ketamine and xylazine mixture (90 mg/kg and 8mg/kg, respectively). SNI in rats was performed following the same procedures as for mice. To create a dorsal column lesion in the spinal cord, a dorsal laminectomy was performed at the T9 level and bilateral dorsal columns with adjacent lateral columns were cut out with iridectomy scissors. To visualize regenerating axons, cholera toxin subunit B (CTB; List Biological Laboratories) was injected using a protocol modified slightly from that in the previous report 21. Briefly, after the sciatic nerve between the thigh muscles was exposed, a small incision was made on the perineurium just proximal to the trifurcation site. Two microliters of unconjugated CTB solution (1% in PBS) were slowly injected using the Hamilton syringe through the perineural incision and animals were killed 5 d after the injection.

### Primary culture of dissociated adult DRG neurons and neurite outgrowth assay

DRGs were freshly dissected and treated with 125 U/ml type XI collagenase (Sigma-Aldrich) dissolved in DMEM (Hyclone) for 90 min at 37°C with a gentle rotation. After washing five times with DMEM, cells were dissociated by trituration using a pipette tip and centrifuged at 1500 rpm for 3 min. Cell pellets were resuspended in Neurobasal-A (Invitrogen) supplemented with B-27 (Invitrogen) and plated onto eight-well culture slides (BD Biosciences) precoated with 0.01% poly-D-lysine (Sigma-Aldrich). The culture slides were incubated with 3 μg/ml laminin (Invitrogen) for 2 h at 37ºC before cell plating. When assessing neurite outgrowth under a growth-inhibitory environment, the culture slides were coated with 1.0 μg/ml chondroitin sulfate proteoglycans (CSPGs; Millipore) together with laminin. The culture duration was strictly limited to 15 h, at which time point only negligible neurite outgrowth is observed in the control condition (without conditioning injury). To compare the potency of ONCM for neurite outgrowth activity with that of other growth-promoting agents, DRG neurons were allowed to grow for 24 h since the previous reports used 24 h culture duration. For treatment of NGF and hyper IL-6, cells were treated with NGF (Life technologies) or hyper IL-6 (R&D systems) for 24 h immediately after cell plating. For co-treatment of NGF and hyper IL-6, cells were treated with NGF (Life Technologies) for 6 h, then treated with hyper IL-6 (R&D system) for 18 h. For treatment of Forskolin (15 μM; Sigma-Aldrich), D-mannose (1 μM; Sigma-Aldrich), dibutyryl-cAMP (db-cAMP; 100 µM; Calbiochem), and 100 ng/ml of recombinant ONCM, cells were treated with forskolin, mannose, and recombinant ONCM or db-cAMP and recombinant ONCM for 24 h immediately after cell plating. Neurite outgrowth was visualized by immunostaining with mouse anti-beta3 tubulin (1:1000; Promega) followed by incubation with Alexa 594-conjugated anti-mouse secondary antibodies (Invitrogen).

### Primary bone marrow-derived macrophage culture

To harvest bone marrow-derived macrophages (BMDMs), adult mice were anesthetized with an overdose of ketamine and xylazine mixture. Bilateral femurs and tibias were flushed using 26-gauge needles into DMEM containing 10% FBS (Hyclone). Bone marrow cells were collected and centrifuged at 1500 rpm for 5 min. Cell pellets were resuspended in red blood cell lysis buffer (0.15 mol/l NH_4_Cl, 10 mmol/l KHCO_3_, and 0.1 mmol/l Na_2_EDTA, pH 7.4). Purified bone marrow cells were centrifuged at 1500 rpm for 3 min and filtered through a 100 μm cell strainer (BD Biosciences). The bone marrow cells were plated onto a 100 mm culture dish or six-well plate (BD Biosciences) coated with 0.01% poly-D-lysine and cultured in DMEM supplemented with 1% penicillin/streptomycin and 10% FBS. Macrophage colony-stimulating factor (M-CSF; Invitrogen) was added at a concentration of 20 ng/ml to facilitate differentiation of the bone marrow cells into macrophages for 7 to 10 days 62.

### Neuron-macrophage co-culture

Neuron-macrophage co-cultures were established following the methods described previously 27 with slight modifications. Dissociated DRG neurons were incubated in a 6-well plate with DMEM supplemented with B-27 for 4 h. Then, BMDMs were plated on the transwell inserts placed on top of the 6-well plate wells at a ratio of 1:5 (neurons to macrophages). Four h after setting up the co-cultures, cells were treated with db-cAMP (100 µM) or PBS as a control. After 24 h, BMDMs grown on the cell culture inserts were transferred to a new culture plate. The culture medium was replaced with fresh DMEM supplemented with B-27. The co-cultures were maintained for 72 h without changing the medium and then MCM was collected, centrifuged at 1500 rpm for 5 min, and passed through a 0.2 µm filter (BD Biosciences) to remove any remaining cellular debris. For neurite outgrowth assays with MCM, primary dissociated DRG neurons were plated on eight-well culture slides as described above and maintained in culture for 2 h to allow for attachment to the culture dishes before replacing the medium with the collected MCM. After 15 h in culture, the cells were fixed and immunostained for beta3 tubulin to visualize neurite outgrowth.

### Neurite outgrowth assay with primary cortical neurons and spinal cord derived neural stem cells (NSCs)

Primary cortical neuron cultures were prepared from embryonic day 17 C57BL/6 mouse embryos. At 7 DIV, cultured neurons were scraped with a 96-well floating pin tool (V&P Scientific) as previously reported 31, 32. After scraping, the whole medium was replaced with a fresh one, and ONCM recombinant protein (100 ng/ml or 1000 ng/ml) was added to the medium. Two days following the scraping, neurons were fixed with 4% paraformaldehyde and stained with anti-beta3 tubulin (1:1000). Primary rat neurospheres were obtained from embryonic day 14 rat spinal cord as previously described 63. Dissociated NSCs from the neurospheres were plated at a density of 1 × 10^4^ cells per coverslip with DMEM containing 3% FBS. Four h later, NSCs were washed with a neurobasal medium and incubated with a differentiation medium containing the neurobasal supplemented with 2% B-27 and 1% GlutaMAX. On the 6^th^ day of neuronal differentiation, ONCM was added to the fresh media at a concentration of 100 nM/ml for 24 h, and the cultured NSCs were fixed with 4% paraformaldehyde and stained with anti-beta3 tubulin (1:1000).

### Quantitative measurement of mRNA by real-time PCR

Total RNAs were extracted from dissected DRGs or cultured cells using Trizol (Invitrogen) according to the manufacturer's protocol. The amount of RNA was determined using NanoDrop Lite Spectrophotometer (Thermo Fisher Scientific) at 260 nm. Two micrograms of RNA was reverse-transcribed to cDNA using a standard RT protocol. One microgram of cDNA was added to the PCR-reaction premix (Takara Bio) with 10 pM corresponding primer pairs. The following primers were used for PCR: 18S ribosomal RNA (rRNA), 5'-CGGCTACCACATCCAAGGAA-3' (forward), 5'-TGCTGGCACCAGACTTGCCCTC-3' (reverse); ONCM, 5'-TTCTGAGCGCTGATGACATT-3' (forward), 5'-CGCTCTGGAACCTCTGTAGG-3' (reverse); NPY, 5'-TGGACTGACCCTCGCTCTAT-3' (forward), 5'-TGTCTCAGGGCTGGATCTCT-3' (reverse); Galanin, 5'-GTGACCCTGTCAGCCACTCT-3' (forward), 5'-GGTCTCCTTTCCTCCACCTC-3' (reverse); Sprr1a, 5'-CCCCTCAACTGTCACTCCAT-3' (forward), 5'- CAGGAGCCCTTGAAGATGAG-3' (reverse); Vip, 5'-TTCACCAGCGATTACAGCAG-3' (forward), 5'-TCACAGCCATTTGCTTTCTG-3' (reverse); Map2, 5'-CTGGACATCAGCCTCACTCA-3' (forward), 5'-AATAGGTGCCCTGTGACCTG-3' (reverse); CD68, 5'-ACTCATAACCCTGCCACCAC-3' (forward), 5'-.GATTTGAATTTGGGCTTGGA-3' (reverse); Glutamine synthetase, 5'-GGGGTGATAGCAACCTTTGA-3' (forward), 5'- ACTGGTGCCTCTTGCTCAGT-3' (reverse); GFAP, 5'-GCTTCCTGGAACAGCAAAAC-3' (forward), 5'-AAGGTTGTCCCTCTCCACCT-3' (reverse); P0, 5'-GGTTTACACGGACAGGGAAA-3' (forward), 5'-GTCCCTTGGCATAGTGGAAA-3' (reverse); S100, 5'-CCATGGAGACCCTCATCAAT-3' (forward), 5'-TTGAAGTCCACTTCCCCATC-3' (reverse). Quantitative RT-PCR was performed according to the protocol supplied by the SYBR Green PCR kit using the 7500 Real-Time PCR System (Applied Biosystems). Cycling conditions were 94°C for 30 s, 55°C ~ 64°C for 30 s, and 72°C for 60 s, with a total of 40 cycles. Melting curves were generated after the last extension step, and the CT values were quantified by the Applied Biosystems 7500 software. Target gene expression was normalized with the expression of 18S rRNA as an internal control.

### ELISA

To quantify the amount of ONCM in DRG tissue, L4 and L5 DRGs were dissected and lysed to extract proteins, and the ONCM concentration was measured using an ELISA kit for oncomodulin (catalog #MBS908312; Mybiosource), according to the manufacturer's protocol. In each well, 50 µl of DRG lysate at a concentration of 100 ng/µl was dispensed. Each experiment was performed in triplicate. To measure the concentration of ONCM in MCM obtained from cultured BMDMs, MCM was centrifuged at 1500 rpm for 5 min and passed through a 0.2 µm filter (BD Biosciences) to remove remaining cellular debris. One hundred microliters of CM was dispensed in each well. Three independent cultures were performed to collect CM for each condition, and each CM was measured in triplicate. To measure the protein level of ONCM in protein lysis buffer treated ONCM conjugated with or without REPL-NG, free recombinant ONCM, ONCM conjugate with REPL-NG, or Glutathione (10 mM; Sigma-Aldrich) was treated with RIPA Lysis buffer (Thermo Fisher) for 10 min, 12 h, or 24 h at room temperature. 50 microliter of each group was dispensed in each well. Four independent experiments were performed for each condition, and each treatment condition was measured in duplicate.

### ONCM expression vector and transfection

pCMV-ONCM was constructed by cloning ONCM cDNA as BamHI/NotI fragment from Myc-DDK-tagged oncomodulin (OriGene) in a mammalian expression vector pCMV (Addgene). To prepare a construct with the signal sequence (SS) for secretion of ONCM by translocation across the endoplasmic reticulum membrane, the N-terminal bacterial alkaline phosphatase (PhoA) signal peptide sequences (MKQSTIALALLPLLFTPVTKAR) were subcloned from pIg20 vector. PhoA signal peptide was amplified using a 5'- ATGAAGCAGTCCACCATCGC PCR primer and 5'- GCGGGCCTTGGTCACGGGGG PCR primer, and the PCR product was subcloned into Strataclone blunt PCR vectors (Stratagene) and subcloned further into pCMV-ONCM to generate the pCMV-SS-ONCM construct. PC12 cells were cultured in DMEM containing 10% horse serum and 1% penicillin-streptomycin. PC12 cells (1.5 × 10^5^ cells) were seeded on PDL-coated 6 well plates and incubated for 24 h. To induce neuronal differentiation, the medium was changed to DMEM containing 1% horse serum, 1% penicillin-streptomycin, and 100 ng/ml NGF. The cells were further cultured for 6 d, and the differentiation medium was replaced every 2 d. HEK293T cells and Raw 264.7 cells were maintained in DMEM supplemented with 10% FBS and 1% penicillin-streptomycin. Cells were transfected with Lipofectamine 2000 (Thermofisher) following the manufacturer's instructions. Briefly, cells were transfected at 60-80% confluency in 6-well plates using 2 μg of the expression plasmid. After 48 hr, cells were washed with PBS and lysed by applying 250 μl lysis buffer (20 mM HEPES at pH 7.5, 100 mM KCl, 5 mM MgCl_2_, 1 mM dithiothreitol, 5% glycerol, and 0.1% Triton X-100 supplemented with Roche Protease Inhibitor cocktail) and then rocked for 10 min at 4°C. The resulting cell lysate was divided into aliquots for further analysis.

### Preparation and injection of recombinant adeno-associated virus

A recombinant adeno-associated virus (AAV) vector containing a CCL2 expression cassette was generated by replacing the GFP cassette of pAAV-CAG-GFP (Addgene) with the full-length cDNA for CCL2 (OriGene). AAV serotype 5 (AAV5) virus preparation was performed at the University of North Carolina at Chapel Hill Vector Core facility. Control AAV5-GFP was purchased from the same facility. The titer of both viruses was ~1 x 10^12^ genome copies/ml. For intraganglionic injection, the L5 DRG was exposed after the removal of the lateral process of the L5 vertebral bone. A Hamilton syringe configured with a glass pipette was controlled using a micromanipulator, and the tip of the glass pipette was slowly advanced into the L5 DRG under a surgical microscope; 2 µl of AAV5-CCL2 or AAV5-GFP was injected into the L5 DRG at a rate of 0.25 µl/min using a nanoinjector (#M3301R, World Precision Instruments). The recombinant AAV vector containing ONCM or SS-ONCM expression cassette was generated from pCMV-ONCM and pCMV-SS-ONCM. AAV5 viral particles containing either ONCM or LS-ONCM were prepared at the Korea Institute of Science and Technology virus facility. The titer of both viral particles was ~1 x 10^14^ genome copies/ml. Intraganglionic injection was done using the same procedures described above.

### Isolation and sequencing of RNA from cultured DRG neurons

Cultured DRGs neurons were established following the methods described above. For further purification, the cell suspension was layered on a BSA cushion (10% w/v in Neurobasal-A) and centrifuged at 600 g for 13 min at a low brake. Myelin debris and lighter non-neuronal cells were settled at the top layer of 10% BSA, whereas the neurons were pelleted down. Cell pellets were resuspended in Neurobasal-A (Invitrogen) supplemented with B-27 (Invitrogen) and plated onto a six-well plate. Immediately after setting up the cultured DRG neurons, cells were treated with 100 ng/ml of recombinant ONCM. RNA was extracted using RNeasy Plus Micro Kit (Qiagen) following the manufacturer's protocol. Purified RNA samples at a concentration of 200-500 ng/µL for 20 µL and RNA Integrity Number (RIN) above 9.5 were processed for library preparation and RNA-sequencing at Macrogen (Seoul, Korea). cDNA libraries were prepared using Trueseq Stranded mRNA Prep Kit (Illumina). The cDNA libraries were then sequenced using the Illumina Novaseq 6000 platform with 150 bp paired-end reads. Gene ontology (GO) analysis for differentially expressed genes (n=167, PBS vs. ONCM, fold difference on log2 scale > 2) was performed by using DAVID software (http:// https://david.ncifcrf.gov). For neuropeptide-related signatures in the GO database, gene set enrichment analysis (GSEA) was performed using GSEA (https://www.gsea-msigdb.org/gsea).

### Synthesis and characterization of reducible ε-poly(_L_-lysine) nanogel (REPL-NG)

REPL-NG was synthesized by a two-step chemical reaction: the thiolation of ε-poly(_L_-lysine)** (**EPL, Zhengzhou Bainafo Bioengineering, China) by 2-iminothiolane (IT, Sigma, USA) and then the formation of multiple disulfide bonds among thiols in thiolated EPL. In detail, after EPL (200 mg, 42.6 μmol) and IT (213 μmol; five equivalents to the moles of EPL) were dissolved in 8 mL and 1 mL of Dulbecco's phosphate-buffered saline (DPBS; pH 7.4), respectively, two reactant solutions were mixed and then reacted to thiolate some primary amines of EPL for 24 h at room temperature. Then, 3 mL of DMSO was added to the thiolated EPL solution to form disulfide bonds, and the reaction solution was stirred for an additional 24 h. After transferring the resulting REPL-NG-containing solution into a dialysis membrane (molecular weight cut-off 25 kDa), the REPL-NG was purified by dialysis against deionized water for 48 h. The dialysate was filtrated to remove unwanted aggregates by a paper filter and was lyophilized. In the resulting REPL-NG, the number of the IT-derived groups was approximately 5.4 per single EPL polymer chain and was detected by a 500 MHz Bruker ^1^H-NMR spectrometer (Bruker, USA). The particle size and zeta-potential of REPL-NG were 11.0 ± 1.4 nm (polydispersity index = 0.224) and 43.9 ± 6.1 mV, respectively, and were monitored by a zeta-sizer (ELS-Z, Photal Otsuka Electronics Co., Japan). The synthesized REPL-NG was stored as a dried solid at -20 ºC prior to use.

### Generation of REPL-NG/ONCM complex and intraganglionic injection of the nanocomplex

Five µg of REPL-NG (5 mg/ml in PBS solution) was mixed with 1.7 µg of recombinant ONCM. To increase electrostatic interaction between recombinant ONCM and REPL-NG, REPL-NG/ONCM complex was subjected to vertical tapping at a weight ratio of 3:1 (REPL-NG to recombinant ONCM). For stabilization of REPL-NG/ONCM complex formation, REPL-NG and ONCM mixture were incubated for 30 min at room temperature. Immediately after the stabilization of REPL-NG/ONCM, 2 µl of REPL-NG/ONCM complex or REPL-NG was injected into the L4 and L5 DRG at a rate of 0.25 µl/min using a nanoinjector (#M3301R, World Precision Instruments). For intraganglionic injection, the L4 and L5 DRGs were exposed after removal of the lateral process of the L4-5 vertebral bone. A Hamilton syringe configured with a glass pipette of which tip diameter was less than 70 µm was slowly advanced into the L4 and L5 DRGs sequentially under a surgical microscope. After injection, the syringe was kept *in situ* for 1 min to prevent regurgitation of the injected REPL-NG or REPL-NG/ONCM complex through the injection site.

### Tissue processing and immunohistochemistry

Rats or mice were anesthetized with ketamine/xylazine mixture and perfused with heparinized PBS followed by 4% PFA in 0.2 M PB. DRGs or spinal cord tissues containing the lesion site were dissected and postfixed in 4% PFA for 2 h, followed by cryoprotection in a graded series of sucrose solutions. DRGs were cryosectioned at 12 µm thickness. For spinal cord tissue, parasagittal cryosections (at 40 μm thickness) were made in a 1:5 series. Tissue sections were mounted onto Super Frost plus slides (Thermo Fisher Scientific) and stored at -20°C until use. DRG sections underwent antigen retrieval with 0.1 M EDTA Tris buffer, pH 9.0, at 98ºC and were treated with 10% normal goat serum and 0.3% Triton X-100 for 1 h, and then the primary antibodies, dissolved in the same blocking solution, were applied at 4°C overnight. The primary antibodies were rabbit anti-GFAP (1:500; Dako), chicken anti-GFP (1:1000; Abcam), goat anti-CTB antibodies (1:10000; List Biological Laboratories), rabbit anti-Iba-1 (1:500; Dako), rabbit anti-galanin (1:1000, Millipore), rabbit anti-c-Myc (1:250, Abcam), rabbit anti-Sprr1a (1:1000; Abcam), rabbit anti-phospho-c-Jun (1:100; Millipore). Tissue sections were washed thoroughly and then incubated with appropriate secondary antibodies tagged with AlexaFluor-488 or -594 (1:500; Invitrogen) for 1 h at room temperature. Some DRG tissue sections were incubated for 30 min with NeuroTrace 640/660 for Deep-Red Fluorescent Nissl stain (1:300; Invitrogen) and underwent three wash steps of 5 min each in PBS. The coverslips were mounted onto slides with a glycerol-based mounting medium (Biomeda). The images were taken using an LSM-800 confocal microscope (Zeiss) and Axio scan Z1 (Zeiss).

### Quantitative image analysis

For neurite outgrowth assay, the mean neurite length per neuron was measured to compare the extent of neurite outgrowth between different experimental conditions. Neurite length was measured using MetaMorph Image Analysis Software (Molecular Devices). Each well was divided into four quadrants, and a 200× magnification image was obtained at the center of each quadrant (four images in each well). Measurements were made in exported eight-bit TIFF files and neurite length was determined as follows: the MetaMorph “draw” function was used to draw a line with neurite and the values were calibrated in micrometers using the MetaMorph “calibrate” function. To quantify the intensity of IBA-1 immunoreactivities, two ROIs each covering 150 mm^2^. All images were obtained using the same detector setting. After images were adjusted using the predetermined threshold setting using ImageJ software (available at: http://imagej.nih.gov/ij/), the integrated intensity of the immunoreactivity per unit area was obtained. To quantify the extent of dorsal column axon growth after injury, two consecutive parasagittal sections 200 μm apart from each other containing CTB-traced axons were used for analysis. After the caudal lesion border was identified using GFAP staining, lines perpendicular to the longitudinal axis were drawn at 200 µm intervals from the lower lesion border to delineate counting blocks, with blocks named according to their shortest distance to the lesion border. CTB positive axon numbers between successive lines were counted and recorded as the number of axons in the block. The values were averaged from the sections with visible axons in each animal. In addition, the longest distance of regenerating axons beyond the caudal lesion border was recorded for each animal.

### Fluorescence-Activating Cell Sorting (FACS) for isolation of macrophages

SNI was performed bilaterally in the Cx3cr1-GFP mouse. Seven days after injury, L3, 4, and 5 DRGs were freshly dissected and treated with 125 U/ml type XI collagenase (Sigma-Aldrich) dissolved in DMEM (Hyclone) for 90 min at 37°C with a gentle rotation. After washing five times with DMEM, cells were dissociated by trituration using a pipette tip and centrifuged at 1500 rpm for 3 min. Cell pellets were resuspended in Neurobasal-A (Invitrogen) supplemented with B-27 (Invitrogen). For further purification, Cell suspension was layered on a 30% BSA and centrifuged at 600 g for 10 min at a low brake and repeat BSA cushion methods 2 more times with 15% BSA and 10% BSA, respectively. Cell pellets were resuspended in HBSS (Hyclone). The GFP positive and negative fractions were collected separately in a 15 ml comical tube based on GFP fluorescence using FACSAria III (Becton Dickinson). Each tube was centrifuged at 1500 rpm for 10 min at 4 °C. Total RNA was extracted from positive and negative fraction cells using the methods described above.

### Statistical analysis

All numerical values and error bars in the quantification graphs are expressed as mean SEM. Statistical comparison of mean values was performed using unpaired Student's t-tests or one-way ANOVA followed by Tukey's *post hoc* tests. Quantification graphs were generated using GraphPad Prism version 8.00 (GraphPad Software).

## Supplementary Material

Supplementary figures.
