# Supplementary material for: Nanogel-mediated delivery of oncomodulin secreted from regeneration-associated macrophages promotes sensory axon regeneration in the spinal cord
Source: Theranostics. 2022 Aug 1;12(13):5856–76. doi: 10.7150/thno.73386 (PMC9373827; doi:10.7150/thno.73386)

Figure S1. Colocalization of Cx3cr1-GFP with IBA1 and flow cytometric analysis.

A, Confocal images of L5 DRG sections doubly stained for GFP (green) and NeuroTrace (violet) at 0 (CTL) and 7 d (7D) after sciatic nerve injury. Scale bars represent 50 µm. B, Representative histogram of the flow cytometry analysis of the GFP fluorescence in dissociated DRG cells from Cx3cr1-GFP mice following sham injury (CTL) and 7 d after injury (7D).

Figure S2. Preconditioning effects are not affected in ONCM-deficient mice at an earlier time point.

A, Representative images of neurite outgrowth of DRG neurons taken from WT and $ONCM^{-/-}$ mice at different time points after sciatic nerve injury. DRG neurons from the L3, L4, and L5 DRGs were dissociated and cultured for 15 h before being fixed for the immunofluorescent visualization of neurites with anti-beta3 tubulin. Scale bars represent 100 µm. B, Comparison of the mean neurite length between cultures from WT and $ONCM^{-/-}$ mice 0 (CTL), 2 (2D), and 7 d (7D) after injury. N = 4 animals for each condition. $^{***}p < 0.001$ by unpaired *t* test.

Figure S3. Comparison of macrophage activation at the injury site and DRG between WT and ONCM deficiency.

A, Representative images of Iba-1-immunostained sciatic nerve sections obtained from animals subjected to 0 (CTL) or 7 d after sciatic nerve injury. Scale bars represent 50 µm. B, Confocal images of the L5 DRG sections obtained from WT and $ONCM^{-/-}$ mice doubly stained for Iba-1 (green) and NeuroTrace (violet) sham injury (CTL) and 7 d after injury (7D). Scale bars represent 50 µm.

Figure S4. Intraganglionic AAV5-CCL2 injection increases the number of macrophages irrespective of the genotype.

A, Representative images of Iba-1 staining in L5 DRG sections obtained from WT and $ONCM^{-/-}$ mice at 28 d after intraganglionic injection of AAV5-GFP or AAV5-CCL2. Scale bars represent 50 μm. B, Comparison of the number of macrophages. N = 6 animals per group. ***$p < 0.001$ between AAV5-GFP and AAV5-CCL2 injection groups by one-way ANOVA followed by Tukey's *post hoc* analysis.

Figure S5. Effects of ONCM on neurite outgrowth in cultured cortical neurons and neural stem cells (NSCs)

A, Representative images of beta3 tubulin staining in cultured cortical neurons. Cortical neurons were scraped at DIV 7, and were allowed to regenerate their neurite for 48 h with or without ONCM. B. Quantification graph of the extent neurite regeneration as expressed percent of the mean control value. N = 3 replicate scrape assays. C. Representative images of beta3 tubulin staining in cultured NSCs derived from embryonic 14 rat spinal cord tissue. NSCs were differentiated into the neuronal lineage for 6 days, after which ONCM was added to the culture for 24 h. D. Quantification graph of the mean length of the beta3 tubulin positive neurites. N = 4 independent cultures.

Figure S6. BSA cushion diminished expression of non-neural cell-specific genes.

Representative images of electrophoresed RT-PCR products of various gene expression from DRG cell fractions purified by with (+) or without (-) BSA cushion method. 18S rRNA was used as an internal reference.

Figure S7. Validation of the neuropeptide gene upregulation in DRG neurons by

ONCM.

A, Representative images of electrophoresed RT-PCR products from cultured DRGs treated with PBS or ONCM. 18S rRNA was used as an internal reference. B, Representative images of electrophoresed RT-PCR products. 18S rRNA was used as an internal reference. DRG samples were obtained 0 (CTL), 1, 3, and 7 d after sciatic nerve injury.

Figure S8. Intraganglionic injection of REPL-NG/ONCM does not induce activation of macrophages or neuronal damages in DRGs.

A, Representative images of DRG sections stained with Iba-1 (green) and NeuroTrace (violet) 14 d after intraganglionic injection of REPL-NG only or REPL-NG/ONCM. DRGs were freshly dissected from animals at 0 (sham), 14 d after SNI (SNI). Scale bars represent 50μm.

Figure S9. Intraganglionic injection of REPL-NG/ONCM upregulates galanin immunoreactivity.

A, Representative images of Galanin-immunostained DRG sections from animals subjected to spinal cord injury (SCI) only or injected with REPL-NG 14 d before injury (REPL-NG; -14D), with REPL-NG/ONCM 14 d before injury (REPL-NG/ONCM; -14 D), or with REPL-NG/ONCM 1 d after injury (REPL-NG/ONCM; +1D), and those subjected to preconditioning SNI before creating the spinal lesion (SNI). Scale bars represent 100 μm.

Supplementary figure 1.

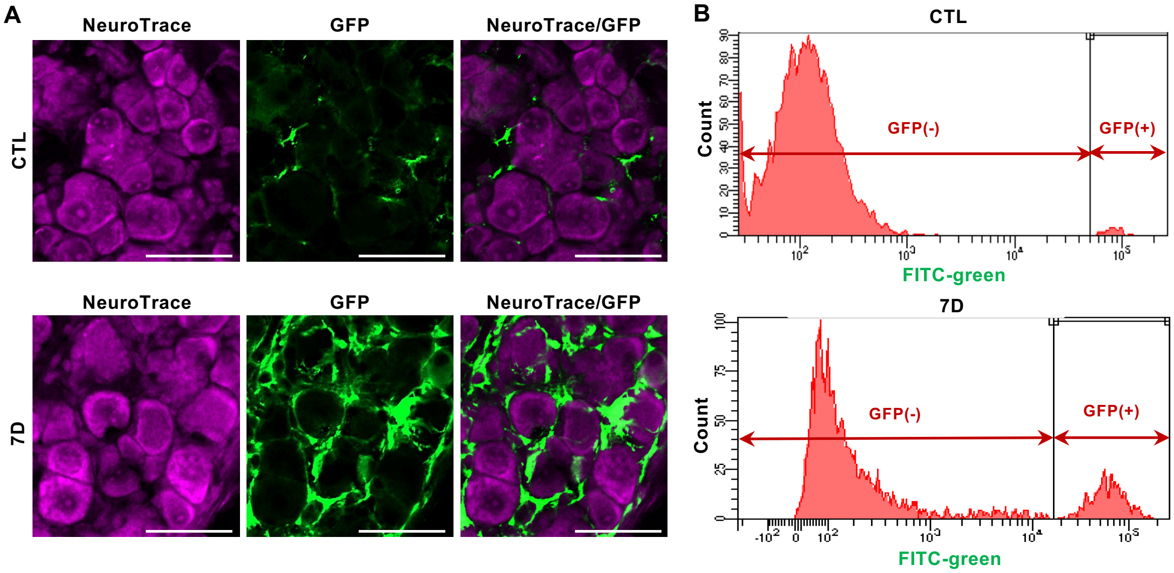

Supplementary figure 2.

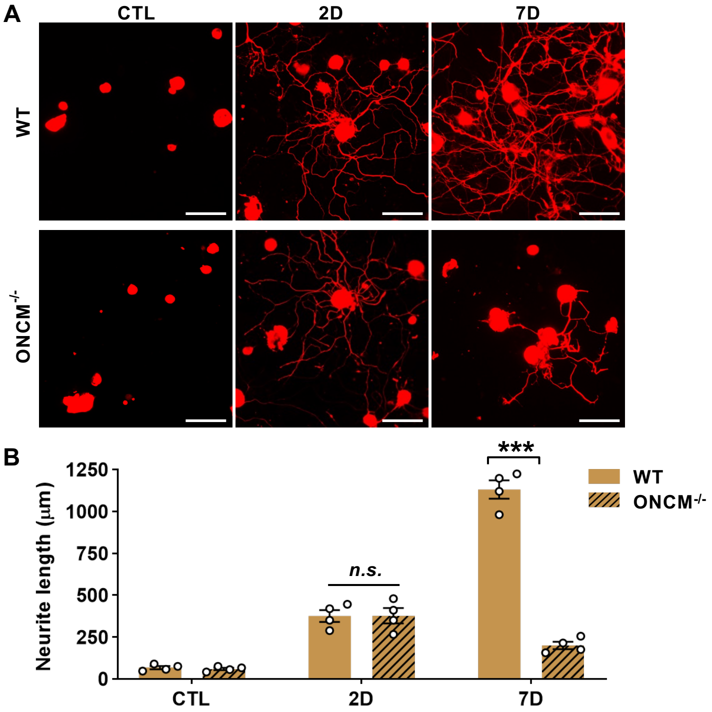

Supplementary figure 3.

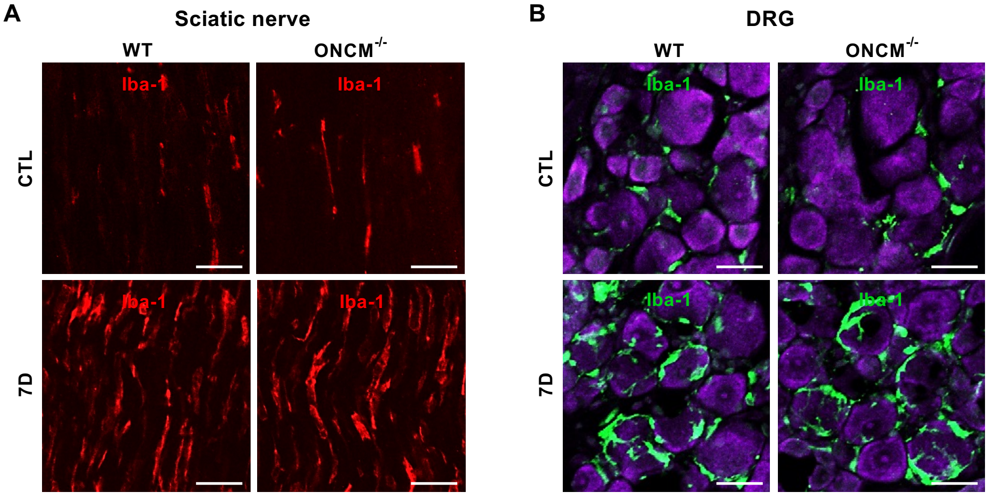

Supplementary figure 4.

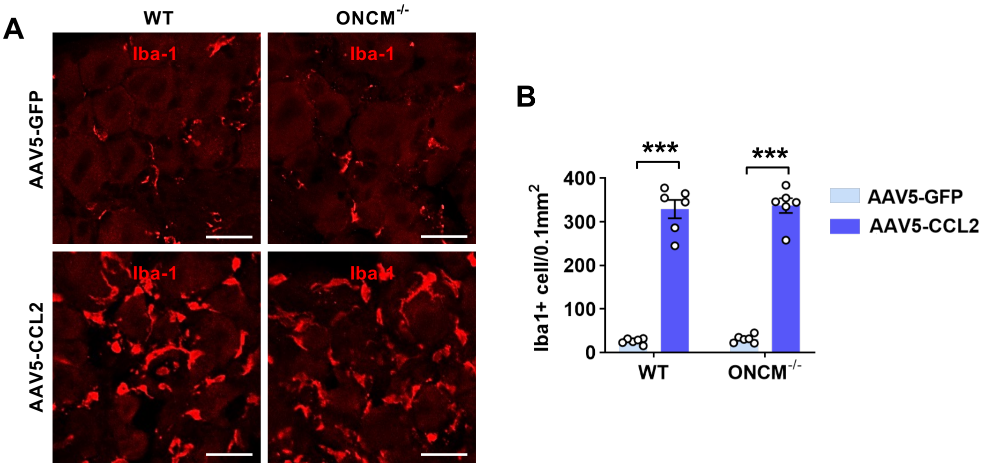

Supplementary figure 5.

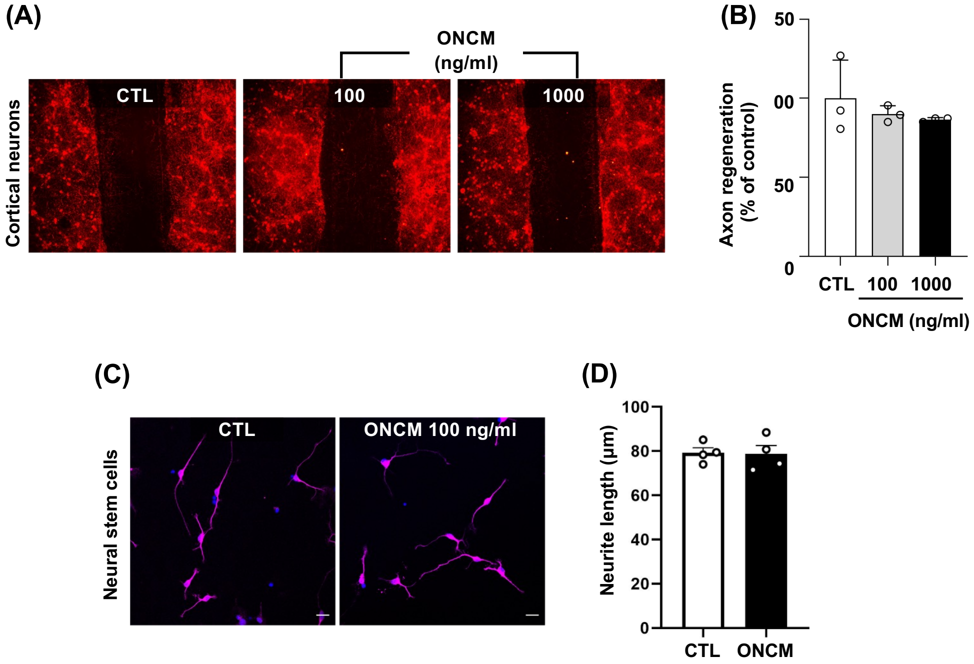

Supplementary figure 6.

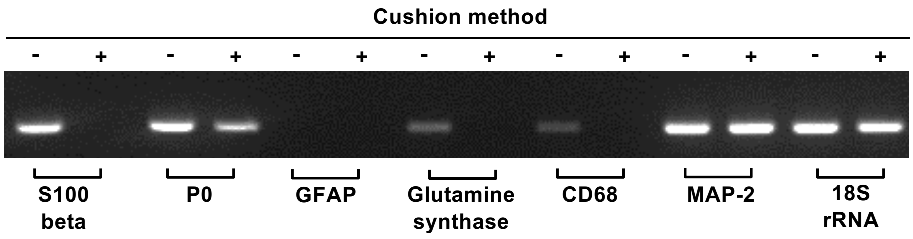

Supplementary figure 7.

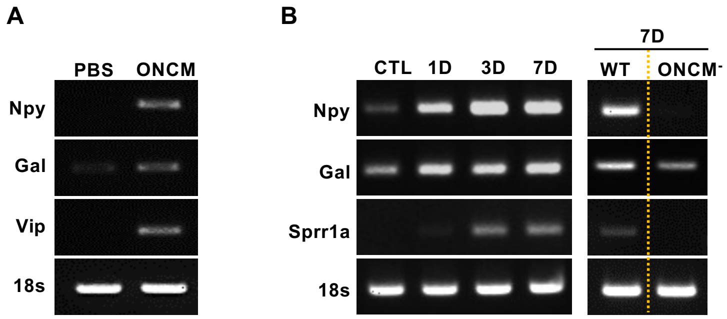

Supplementary figure 8.

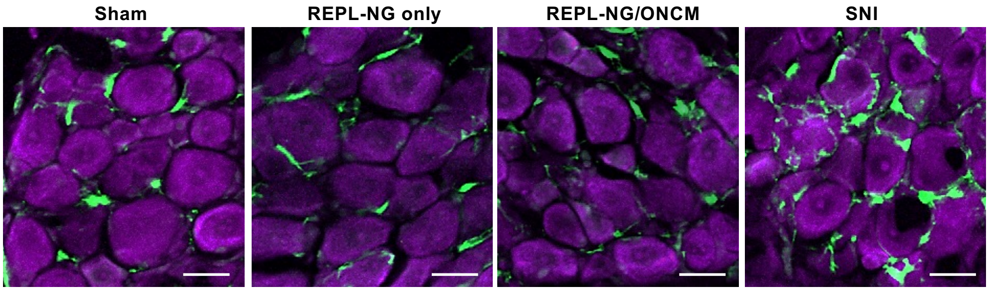

Supplementary figure 9.

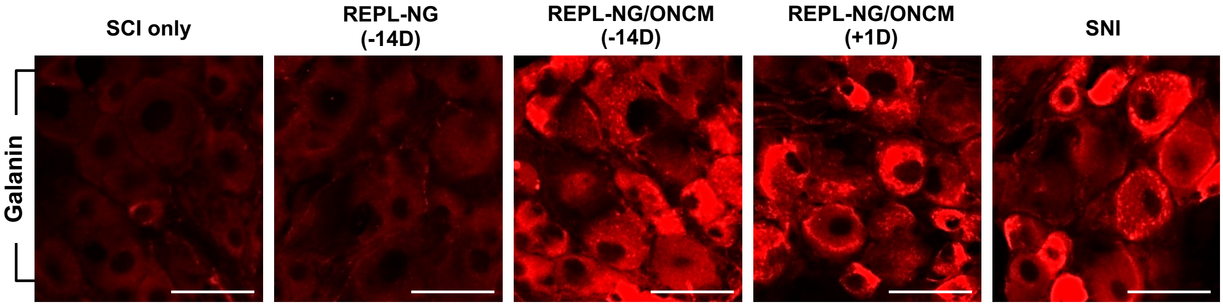

Supplement: Supplementary file 1 — Supplementary figures. [file thnov12p5856s1.pdf]
